# Exposures to Drinking Water Contaminants in Community Water Systems and Risk of Ovarian Cancer in the California Teachers Study Cohort

**DOI:** 10.1021/EHP.6c00169

**Published:** 2026-05-20

**Authors:** Maya Spaur, Lauren M. Hurwitz, Danielle N. Medgyesi, Alexander P. Keil, Laura E. Beane Freeman, Jared A. Fisher, Jessica M. Madrigal, Samantha Ammons, Emma S. Spielfogel, Komal Bangia, Paul S. Albert, Tiffany R. Sanchez, James V. Lacey, Rena R. Jones, Mary H. Ward

**Affiliations:** † Occupational and Environmental Epidemiology Branch, Division of Cancer Epidemiology and Genetics, 682447 National Cancer Institute, Rockville, Maryland 20892, United States; ‡ Department of Environmental Health Sciences, Mailman School of Public Health, 5798 Columbia University, New York, New York 10032, United States; § Division of Health Analytics, Department of Computational and Quantitative Medicine, Beckman Research Institute City of Hope, Duarte, California 91010, United States; ∥ Community and Environmental Epidemiology Research Branch, Office of Environmental Health Hazard Assessment, Oakland, California 94612, United States; ⊥ Biostatistics Branch, Division of Cancer Epidemiology and Genetics, 3421 National Cancer Institute, Rockville, Maryland 20850, United States; # Department of Epidemiology, Colorado School of Public Health, Colorado University Anschutz, Aurora, Colorado 80045, United States

## Abstract

**BACKGROUND**: Several drinking water contaminants are known or suspected carcinogens; however, there are only a few investigations of drinking water exposure and ovarian cancer. We evaluated associations between regulated contaminants in community water systems (CWS) and ovarian cancer risk in the California Teachers Study, a prospective cohort of female California educators. **METHODS**: Participants were cancer-free, without bilateral oophorectomy, living in California at baseline (1995–1996) with geocoded addresses linked to a CWS (*N* = 91,127, 92%), with follow-up through 2020 (mean = 19.0 years). Among participants with a residential duration at enrollment of at least 10 years, we computed 15-year (1990–2005) averages of log2-transformed arsenic, nitrate, total trihalomethanes (TTHM) (*N* = 59,881), and uranium concentrations (*N* = 56,314). We estimated hazard ratios (HRs, 95% CIs) for all epithelial ovarian cancers (*n* = 413) and the high-grade serous histotype (*n* = 199), using Cox proportional hazards regression, adjusting for age, body mass index, menopause status, oral contraceptive use, and parity. We evaluated the mixture effect (per IQR in log2 concentrations) using quantile-based g-computation. **RESULTS**: Almost all women (>99%) had average exposures below regulatory limits for all contaminants. In single contaminant analyses, a doubling in average uranium concentrations was associated with all ovarian cancer (HR_perlog2_ = 1.07, CI 1.00–1.15), whereas a doubling in nitrate was associated with the high-grade serous histotype (HR_perlog2_ = 1.09, 95% CI 1.01, 1.17). Findings were similar in models adjusted for other contaminants. We observed positive but imprecise associations for arsenic and TTHM in single-contaminant and contaminant-adjusted analyses. HRs per increase in the mixture were 1.34 (0.95, 1.88) and 1.77 (1.09, 2.87) for all ovarian cancer and the high-grade serous histotype, respectively. Uranium was the largest contributor (56%) to the mixture effect for all ovarian cancers, and nitrate was the largest contributor (48%) for the high-grade serous histotype. **CONCLUSIONS**: Novel associations between drinking water contaminants and ovarian cancer risk at levels below regulatory limits warrant further investigation.

## 1 Introduction

Ovarian cancer is the third most common gynecological cancer diagnosed globally and the most fatal gynecologic cancer.^1–3^ Racial and ethnic, socioeconomic, and regional disparities in both incidence and survival in the U.S. have been documented.^4,5^ Ovarian cancer risk increases with age and with genetic (family history, inherited genetic mutations, e.g., *BRCA1* and *BRCA2*), gynecologic (endometriosis), hormonal (use of hormone replacement therapy after menopause), and reproductive (older age at first full-term pregnancy) risk factors.^6^ Protective factors include use of oral contraceptives and having at least one full-term pregnancy.^6^

Drinking water can be a major source of exposure to toxicants, including known or suspected carcinogens. In the U.S., approximately 90% of the population is served by community water systems (CWS).^7^ CWS are regulated by the U.S. Environmental Protection Agency (EPA) under the Safe Drinking Water Act through enforceable maximum contaminant levels (MCLs), standards that consider economic feasibility, technical feasibility/treatment technologies, and public health benefit for certain health end points.^8^ Regulated contaminants fall under six classes, including metals/metalloids (arsenic), radionuclides (uranium and gross alpha), inorganic chemicals (nitrate), and disinfection byproducts (DBPs), such as trihalomethanes (THMs, which include chloroform, dibromochloromethane, bromodichloromethane, and bromoform, and are regulated as the sum total, TTHM).^9,10^ With limited prior epidemiologic evidence, ovarian cancer was not considered in the formulation of existing MCLs.

A positive association was previously observed between drinking water nitrate exposure and ovarian cancer in the Iowa Women’s Health Study, a prospective population-based cohort of postmenopausal women, and in the Agricultural Health Study, a prospective cohort of pesticide applicators and their spouses in Iowa and North Carolina.^11,12^ Nitrate can be ingested from drinking water as well as dietary sources, and is classified by the International Agency for Research on Cancer (IARC) as a probable human carcinogen under conditions that result in the endogenous formation of N-nitroso-compounds (NOCs).^13^

Few other cohort or case-control studies have evaluated drinking water contaminants and ovarian cancer risk.^11,14,15^ Animal studies of drinking water uranium exposure observed uranium accumulation and DNA hypomethylation, a process linked to carcinogenesis, in the ovaries.^16^ Animal evidence also suggests that ingestion of inorganic arsenic may induce ovarian tumors in the offspring of exposed mice.^17^ Uranium is classified as a probable human carcinogen by IARC based on limited evidence for lung cancer, and arsenic is classified as a known human carcinogen based on sufficient evidence for bladder, skin, and lung cancers.^18–20^ Certain THMs have been found to have endocrine-disrupting properties, a mechanism that may induce ovarian carcinogenesis;^21,22^ however, the epidemiologic evidence for an association between drinking water THM exposures and ovarian cancer is inconsistent.^11,12^

Our *objectives* were to evaluate associations between CWS nitrate, uranium, arsenic, and TTHM exposures with ovarian cancer incidence in the California Teachers Study (CTS), a prospective cohort of female California teachers and administrators. We aimed to evaluate the effect of single and co-occurring contaminants at levels of exposure experienced by general U.S. populations. To our knowledge, this is the first analytic epidemiologic study to evaluate the effects of co-occurring nitrate, uranium, arsenic, and TTHM exposures and ovarian cancer risk in U.S. women.

## 2 Methods

### 2.1 Study Population

The CTS is a prospective cohort of women that was designed to investigate the etiology of breast and other cancers.^23^ Public school teachers and administrators in the California State Teachers Retirement System were mailed a self-administered questionnaire; 133,470 completed the survey and enrolled in 1995–1996.^23^ We excluded participants who lived outside of California at baseline (*N* = 8,332), consented to breast cancer research only (*N* = 18), had a prevalent cancer of any type as reported to the California Cancer Registry (*N* = 13,601; does not include nonmelanoma skin cancer), were censored on or before the start date (*N* = 3), had a risk-eliminating surgery (bilateral or second oophorectomy, *N* = 11,786) or did not provide information on a prior oophorectomy (*N* = 796), for a total of 98,934 participants eligible for inclusion in this study (Figure S1). Participants were censored at the earliest of their dates of move out of California, any cancer diagnosis, a bilateral oophorectomy, death, or the end of follow-up (December 31, 2020).

Participants provided information at enrollment (1995–1996) on sociodemographic characteristics (race and ethnicity, age) anthropometrics (height and weight were used to calculate body mass index [BMI], kg/m^2^), smoking and alcohol consumption, and personal medical history (menopause status, oral contraceptive use, total number of live births); educational attainment was collected at questionnaire 4 (2005–2008; response rate 69%). Dietary intake of frequently consumed foods was collected at enrollment via a 1995 103-item Block food-frequency questionnaire (FFQ), which was validated in the CTS.^24,25^ Total daily caloric intake (kilocalories/day), total intake of vitamin C (mg/day) from diet and supplements, and total red meat intake (g/day) were calculated as previously described.^25^ Participants who had missing or unrealistic daily caloric intakes (<600 or >5,000 total kilocalories/day, including alcohol) were excluded from the dietary analyses.^26^ Nitrate and nitrite contents of foods that contributed to each FFQ item were estimated from the published literature.^27^ We computed the nitrate and nitrite intake for each FFQ item (mg) by weighing the food-specific values by sex-specific intake amounts from the 1994–1996 Continuing Survey of Food Intake by Individuals (CSFII)^28^ and multiplying by the reported intake (g/day). We summed across line items to calculate the daily nitrate and nitrite intake overall (milligrams per day) and from plant and animal sources separately, including from processed meats only. Census block group-level characteristics of the enrollment addresses (quartiles of neighborhood socioeconomic status (SES), and urbanicity dichotomized as metropolitan and nonmetropolitan areas) were previously developed and described using 1990 Census data.^24^

### 2.2 Ovarian Cancer Ascertainment

First primary incident ovarian cancers were identified through linkage with the California Cancer Registry, and mortality information was obtained from linkage with the California state mortality file and the Social Security Administration death master file. Epithelial ovarian cancer was defined based on the International Classification of Diseases for Oncology, Third Edition, site C569 and included the following histologic types (histotypes): high-grade serous (*N* = 199), low-grade serous (*N* = 12), endometroid (*N* = 35), mucinous (*N* = 29), clear cell (*N* = 25), and other epithelial (*N* = 113). We evaluated risk for all epithelial ovarian carcinomas (*N* = 413) (hereafter all ovarian cancer) and high-grade serous cases, the most common histotype, separately.

### 2.3 Drinking Water Data and Exposure Assessment

As previously described, we obtained a geospatial data set of statewide drinking water boundaries (the Water Boundary Tool) that was cleaned and processed by the California Office of Environmental Health Hazard Assessment (OEHHA).^29–32^ Monitoring data (1990–2020) for CWS (nitrate, uranium, gross alpha, arsenic, TTHM) were obtained from OEHHA,^29,33^ and average annual concentrations of each contaminant were computed for each CWS. DBP (i.e., THM) measurements were extracted from post-treatment sample points collected within the distribution systems. For all other contaminants, measurements from samples of treated and delivered drinking water were prioritized. Except for the DBPs, when treated samples were not available for a contaminant, we averaged results from raw and untreated samples.^29^ Uranium concentrations were converted from pCi/L to μg/L using 1.49 as the conversion factor (pCi/L*1.49 = μg/L). CWS are required to report nondetections and concentrations when above the detection limits for the purposes of reporting (DLR) (Table S1). Samples marked as “below the DLR”, or with concentrations of zero, or values unlikely to reflect true concentrations (i.e., equal to DLR or 1/2 DLR), were assigned a value based on a single imputation that used Tobit regression, existing measurement data, and assumed a log-normal distribution.^34^ The upper bound for imputation was derived from the median of reported concentrations below the DLR.^34^ Among CWS linked to the enrollment addresses of CTS participants, the *N* (%) that reported ≥1 year of detectable data (not imputed) out of CWS that reported at least one year of measurement data (including imputed values) from 1990 to 2020, was as follows: arsenic (1,163 out of 1,227 CWS; 95%); uranium (818 out of 901 CWS; 91%); gross alpha (1,149 out of 1,224 CWS; 94%); nitrate (1,130 out of 1,232 CWS; 92%); and TTHM (1,229 out of 1,239 CWS; 99%).^32^ Information on CWS water source type (groundwater, groundwater under the influence of surface water, surface water) and CWS size of the population served (very small (≤500 people), small (>500–3300 people), medium (>3300–10,000 people), large (>10,000–<1,000,000 people), and very large (≥1,000,000 people) were also obtained from OEHHA.^32^

Residential histories were previously constructed for CTS participants.^35^ Briefly, addresses at enrollment (1995–1996) were self-reported at the baseline questionnaire, and addresses through 2019 were obtained from participants who completed follow-up questionnaires as well as from the U.S. Postal Service, LexisNexis, Experian, and California Cancer Registry linkages. Self-reported information about years lived at the current address were provided in the fourth (2005–2008; response rate 69%), fifth (2012–2015; response rate 61%), and sixth (2017–2019; response rate 43%) questionnaires.^32^ All participants eligible for inclusion in our study had a geocoded address within CA at baseline. Geocoded addresses were linked to CWS distribution boundaries using QGIS Desktop 3.8.1 and assigned to the intersecting CWS (*N* at enrollment = 91,127, 92%) (Figure S2).^29,32,33,35,36^ Participants whose enrollment address did not link to a CWS (*N* = 7,807, 8%) were assumed to be domestic well users and were excluded from the study (Figure S1).

In our main analyses, we computed 15-year average concentrations (1990–2005) of CWS exposures linked to the address at enrollment and restricted our analyses to participants with a total residential duration at the enrollment address of at least 10 years (based on their self-reported information about residential duration collected at the fourth questionnaire or their residential history), excluding participants on a CWS who resided less than 10 years at the enrollment address (*N* = 27,809). Additionally, we excluded participants who were missing CWS arsenic, nitrate, TTHM, and gross alpha estimates (*N* = 3,437), for a total of 59,881 participants (total person-years = 1,139,582; mean follow-up = 19.0 years) (Figure S1). A subset of these participants also had CWS uranium estimates (*N* = 56,314, 94%; total person-years = 1,072,419, mean follow-up = 19.0 years). We also computed the percent of years in 1990–2005 that annual average CWS concentrations were ≥1/2 the MCL, out of the years of monitoring data reported for each CWS (1990–2005). We used the current MCLs for arsenic (10 μg/L, established by the Final Arsenic Rule, effective 2006; the prior MCL for arsenic was 50 μg/L), uranium (30 μg/L, established by the Radionuclides Rule, effective 2003), gross alpha (15 pCi/L, established by the Radionuclides Rule), nitrate-nitrogen (NO_3_-N, hereafter referred to as nitrate; 10 mg/L, established by the Chemical Contaminant Rules, effective 1992), and TTHM (80 μg/L, established by the Stage 1 and 2 Disinfectants and Disinfection Byproducts Rules, enacted 2006, effective for all CWS in 2013).^10,37–41^ We performed Spearman correlation analyses to describe the correlations between contaminant concentrations.

In additional analyses, we linked participants’ addresses from their postenrollment residential history that included residential changes over follow-up to the corresponding CWS (86,294; Figure S1) and the yearly average estimates of CWS exposures for each year at the residence(s). We interpolated data for years without measurements using nine-year fixed compliance cycles established by the U.S. EPA under the Standardized Monitoring Framework; each compliance cycle is further divided into three monitoring periods of 3 years each.^40^ We interpolated data using the average of annual concentrations from the corresponding monitoring period, and if unavailable, we substituted the average estimate from the (1) earlier monitoring period or, if unavailable, (2) later monitoring period, within the compliance cycle (Table S2).

### 2.4 Statistical Analyses

All statistical analyses were conducted in R version 4.3.3 within the CTS Researcher Platform.^42^ To interpret the results, we considered the magnitude of effect estimates and corresponding 95% confidence intervals (CI) and statistical significance (*p* < 0.05).^43^ We described participant characteristics and CWS contaminant exposures overall and among all ovarian cancer cases and the high-grade serous histotype. We used Cox proportional hazards regression models, with time-on-study as the time scale, to estimate hazard ratios (HR) and 95% CIs for the associations between drinking water contaminant exposures and incident ovarian and high-grade serous cancers.

In models of the 15-year average concentrations, we parametrized drinking water exposures in two ways: continuously after a base-2 log transformation and in categories defined by quantiles (0–<25% [reference], 25–<50%, 50–<75%, 75–<90%, ≥90%). In categorical analyses, we tested for a linear trend using the median of each quantile parametrized as a continuous variable. In models of the percent (%) of years in 1990–2005 that annual average concentrations were ≥1/2 of the MCL, we evaluated categories of >0–≤10% and >10% of years compared to 0% (reference). Models were adjusted for potential confounders and other known risk factors: baseline age and baseline age^2^ (*model 1*), and further adjusted for BMI category (<25 kg/m^2^, 25–<30 kg/m^2^, ≥30 kg/m^2^, or missing), menopause status (pre-, peri/postmenopause, or missing), ever had live births (yes/no/missing), and oral contraceptive use (ever/never/missing) (*model 2*).^44–46^ In our main analyses, we focused on CWS nitrate, uranium, arsenic, and TTHM exposures. We evaluated gross alpha exposures in supplemental analyses to compare to the findings for uranium, as more CWS provided gross alpha monitoring data than uranium due to state primacy regulations. In California, CWS were permitted by Section 64442­(f) of Title 22 of the California Code of Regulations to substitute gross alpha activity measurements for uranium measurements if the gross alpha concentration did not exceed 5 pCi/L.^29^ We additionally evaluated potential nonlinearity in the associations using cubic splines.

To evaluate the joint effects of the drinking water contaminant mixture on incident ovarian and high-grade serous cancer, we used quantile-based g-computation via the *qgcomp* package in R.^47,48^ Nitrate, uranium, arsenic, and TTHM estimates were log2 transformed and divided by the interquartile range (IQR) to standardize the concentrations. We estimated the hazard ratios per IQR increase in the four-contaminant mixture and evaluated the contribution of each contaminant to the overall effect. We explored mixtures composed of different combinations of co-occurring contaminants (composed of two or three contaminants) that were adjusted for the contaminants not included in the mixture. Separately, we evaluated models that were adjusted for all other contaminants.

In time-varying analyses, cumulative average concentrations were lagged 5 years. We evaluated the adjustment for age as a time-varying covariate in these models; other covariates were otherwise identical to the main analyses.

We explored potential effect modification by menopausal status and BMI category. Ovarian cancer is more commonly diagnosed among postmenopausal women than premenopausal women, as the risk of developing ovarian cancer increases with age.^1^ Obesity has estrogenic effects due to the aromatization of androgens in adipocytes, which is associated with an increased risk of development and progression of breast and other cancers.^49–52^ Some evidence suggests that arsenic and uranium may be obesogenic.^53,54^ We also evaluated potential effect modification by smoking status, as cigarette smoking is a source of exposure to arsenic, tobacco-specific nitrosamines, and radioactive elements.^55,56^ We stratified and mutually adjusted analyses by urbanicity (metropolitan and nonmetropolitan areas), CWS water source type, and CWS size, due to differences in contaminant exposures by these characteristics in this cohort.^32^ We used the likelihood ratio test to determine statistical heterogeneity for all stratified analyses, deriving a p-value from the Chi squared test statistic.

We evaluated associations between CWS nitrate and ovarian cancer risk stratified by tertile of daily vitamin C intake and red meat intake (the major source of heme iron) based on previous evidence suggesting modification of cancer risks by these factors due to their role in decreasing and increasing endogenous nitrosation, respectively.^11,13^ We also conducted exploratory analyses evaluating associations with arsenic and uranium exposure stratified by tertile of daily vitamin C intake, based on limited evidence that vitamin C intake may decrease metal/metalloid toxicity.^57^ In supplemental analyses, we also evaluated associations with total dietary nitrate and nitrite as well as plant, animal, and processed meat nitrite sources separately.

### 2.5 Sensitivity Analyses

Because most exposure data were postenrollment (15-year averages at the 1995–1996 enrollment address were calculated using 1990–2005 data), and overlapped with the first ten years of follow-up, we performed a sensitivity analysis in which we started follow-up on January 1, 2005, and excluded participants who were censored before that time. Separately, to confirm whether the observed associations were consistent when all exposures were below regulatory limits, we evaluated single contaminant models and mixtures analyses among participants with average levels (1990–2005) of all contaminants (nitrate, uranium, gross alpha, arsenic, and TTHM) < MCL only.

In a posthoc analysis, we evaluated ovarian cancer risk with time-varying exposures (cumulative average exposures lagged 5 years linked to the residential history) among the same participants that were included in our main analyses (enrollment address duration ≥ 10 years), allowing direct comparisons between findings from the 15-year averages and the time-varying exposures.

## 3 Results

The median baseline age of all participants was 51 years and 54 years for those who developed ovarian cancer (Table 1). Approximately 42% of participants were premenopausal and 48% were peri or postmenopausal (10% missing), while 33% and 56% of participants who developed ovarian cancer were pre or peri/postmenopausal at baseline. Most participants were non-Hispanic white (85%), followed by Hispanic (5%), Asian (4%), black (3%), Native American (1%) and other/multiracial (1%); whereas a higher percent (92%) of cases were non-Hispanic white women. Most participants had a BMI < 25 kg/m^2^, were never smokers, consumed <20g alcohol/day, had a bachelor’s degree or higher, and lived in census block groups in the upper two quartiles of SES and in metropolitan areas at enrollment. A higher proportion of cases had never used oral contraceptives (41% and 39% for all ovarian cancers and the high-grade serous histotype, respectively) compared to the overall cohort (31%).

**Table 1:** Descriptive Statistics of California Teachers Study Participants, Overall and among Participants Who Developed All Epithelial Ovarian Carcinomas and the High-Grade Serous Histotype^a^

	All	All Epithelial Carcinomas	High-Grade Serous
**N**	59881	413	199
**Age, years, Median (IQR)**	51 (44,62)	55 (47,65)	54 (49,65)
**Race and ethnicity, N (%)**			
Non-Hispanic White	51116 (85)	380 (92)	182 (91)
Hispanic	2760 (5)	10 (2)	7 (4)
Asian	2546 (4)	9 (2)	4 (2)
Black	1809 (3)	5 (1)	2 (1)
Other/multi-racial	710 (1)	4 (1)	2 (1)
Native American	425 (1)	1 (0)	1 (1)
Not reported	515 (1)	4 (1)	1 (1)
**N Live Births, Median (IQR)**	2 (1,3)	2 (0.2,2)	2 (1,3)
**Menopause status, N (%)**			
Pre Menopause	25349 (42)	136 (33)	62 (31)
Peri/Post Menopause	28699 (48)	231 (56)	112 (56)
Missing	5833 (10)	46 (11)	25 (13)
**BMI category, N (%)**			
<25 kg/m^2^	34878 (58)	239 (58)	110 (55)
25-<30 kg/m^2^	14455 (24)	104 (25)	56 (28)
≥30 kg/m^2^	8216 (14)	59 (14)	27 (14)
Missing	2332 (4)	11 (3)	6 (3)
**Oral Contraceptive use, N (%)**			
Never	18515 (31)	170 (41)	77 (39)
Ever	39416 (66)	233 (56)	118 (59)
Missing	1950 (3)	10 (2)	4 (2)
**Smoking status, N (%)**			
Never	39731 (66)	267 (65)	128 (64)
Former	16818 (28)	130 (31)	65 (33)
Current	2976 (5)	14 (3)	6 (3)
Missing	356 (1)	2 (1)	0 (0)
**Daily alcohol consumption, N (%)**			
0 g/day	19160 (32)	126 (30)	60 (30)
<20 g/day	33081 (55)	244 (59)	122 (61)
≥20 g/day	4721 (8)	32 (8)	16 (8)
Missing	2919 (5)	11 (3)	1 (1)
**SES** ^b^ **Quartile, N (%)**			
SES 1 (low)	2095 (3)	8 (2)	2 (1)
SES 2	8921 (15)	55 (13)	28 (14)
SES 3	19581 (33)	143 (35)	64 (32)
SES 4 (high)	28942 (48)	204 (49)	103 (52)
Missing	342 (1)	3 (1)	2 (1)
**Urbanicity, N (%)**			
Not metropolitan	18775 (31)	128 (31)	56 (28)
Metropolitan	40777 (68)	282 (68)	141 (71)
Missing	329 (1)	3 (1)	2 (1)
**CWS concentration** ^c^ **, median (IQR)**			
Nitrate-N, mg/L	0.57 (0.22,2.25)	0.66 (0.22,2.20)	0.81 (0.25,2.44)
Uranium, μg/L	3.80 (1.35,6.54)	4.44 (1.44,7.35)	4.31 (1.35,7.54)
Gross alpha, pCi/L	2.15 (1.17,3.49)	2.31 (1.26,3.68)	2.23 (1.19,3.45)
Arsenic, μg/L	0.99 (0.59,1.80)	1.02 (0.6,1.80)	1.14 (0.59,1.84)
TTHM, μg/L	4.56 (0.31,20.84)	5.16 (0.34,21.49)	5.16 (0.50,20.84)

a Community water system (CWS) exposures were linked to the address at enrollment. Analyses were restricted to participants with a residential duration at enrollment ≥10 years[Table-fn t1fn1] (N = 59,881).

b Residential duration at the enrollment address was determined based on the residential history and self-reported information about duration at the current home at questionnaire 4.

c SES = socioeconomic status.

d CWS concentrations represent 15-year average (1990-2005) concentrations.

Medians and interquartile ranges (IQRs) of average CWS exposures were below regulatory limits for all contaminants. We observed modest differences with overlapping distributions in nitrate, uranium, arsenic, and TTHM concentrations comparing all participants to those who developed ovarian cancer (Table 1). Nitrate, arsenic, and uranium concentrations were positively correlated in pairwise analyses (Spearman’s rho ranging from 0.32 for nitrate and arsenic to 0.46 for uranium and arsenic) and negatively correlated with TTHM concentrations (ranging from −0.13 for uranium to −0.33 for nitrate) (Figure S3). Uranium and gross alpha concentrations were highly correlated (0.82).

We describe the findings from the fully adjusted analyses (model 2), which were similar to model 1 results (Table 2). We found positive associations with ovarian cancer (HR, 95% CI) per doubling in average (1990–2005) uranium concentrations (HR = 1.07, 95% CI 1.00, 1.15), with the highest risk for the 90th percentile compared to the lowest quartile (HR= 1.53, 95% CI 1.08, 2.17; p trend = 0.04). A doubling in average nitrate levels was associated with higher risk of high-grade serous cancer (HR = 1.09, 95% CI 1.01, 1.17), with the greatest risk observed at ≥90th percentile of nitrate exposure (HR = 1.54, 95% CI 0.94, 2.54; p trend = 0.03). A doubling in average arsenic exposure was positively associated with total (HR = 1.05, 95% CI 0.96, 1.14) and high-grade serous (1.07, 95% CI 0.95, 1.20) ovarian cancer. No associations were observed with TTHM exposures. Exposure-response models using cubic splines were generally consistent with these findings (Figures 1 and 2).

**Table 2:** Hazard Ratio (95% CI) for All Epithelial Ovarian Carcinomas and the High Grade Serous Histotype per Doubling, and Quantile of Community Water System (CWS) Exposures among California Teachers Study Participants^a^

			All Epithelial Carcinomas			High-Grade Serous Histotype		
	Concentration Range	N	N cases	HR (95% CI) Model 1^c^	HR (95% CI) Model 2^d^	N Cases	HR (95% CI) Model 1^c^	HR (95% CI) Model 2^d^
**Nitrate-N (mg/L)**	log2	59881	413	1.03 (0.98, 1.08)	1.03 (0.98, 1.08)	199	1.09 (1.02, 1.17)	1.09 (1.01, 1.17)
0−<25%	0.01, 0.22	14721	94	1.0 (reference)	1.0 (reference)	41	1.0 (reference)	1.0 (reference)
25−<50%	0.22, 0.56	14759	99	1.05 (0.79, 1.39)	1.04 (0.79, 1.39)	40	0.97 (0.63, 1.50)	0.97 (0.63, 1.49)
50−<75%	0.57, 2.24	15416	118	1.24 (0.95, 1.63)	1.24 (0.94, 1.62)	58	1.4 (0.94, 2.09)	1.38 (0.92, 2.06)
75−<90%	2.25, 3.74	8978	63	1.14 (0.83, 1.57)	1.12 (0.82, 1.55)	35	1.45 (0.92, 2.28)	1.43 (0.91, 2.25)
≥90%	3.77, 9.62	6007	39	1.07 (0.74, 1.56)	1.06 (0.73, 1.55)	25	1.58 (0.96, 2.59)	1.54 (0.94, 2.54)
P trend^e^				0.70	0.74		0.02	0.03
**Uranium (μg/L)**	log2	56314	392	1.08 (1.01, 1.15)	1.07 (1.00, 1.15)	194	1.05 (0.95, 1.15)	1.04 (0.95, 1.15)
0−<25%	0.09, 1.35	13752	81	1.0 (reference)	1.0 (reference)	43	1.0 (reference)	1.0 (reference)
25−<50%	1.35, 3.79	14375	98	1.21 (0.90, 1.63)	1.21 (0.90, 1.62)	51	1.19 (0.79, 1.78)	1.18 (0.78, 1.77)
50−<75%	3.80, 6.46	14103	108	1.37 (1.03, 1.83)	1.36 (1.02, 1.82)	49	1.17 (0.78, 1.77)	1.16 (0.77, 1.75)
75−<90%	6.54, 10.96	8175	54	1.15 (0.81, 1.62)	1.14 (0.81, 1.62)	26	1.04 (0.64, 1.70)	1.03 (0.63, 1.68)
≥90%	11.15, 100.93	5909	51	1.56 (1.10, 2.21)	1.53 (1.08, 2.17)	25	1.44 (0.88, 2.36)	1.42 (0.86, 2.32)
P trend^e^				0.03	0.04		0.26	0.29
**Arsenic (μg/L)**	log2	59881	413	1.05 (0.97, 1.14)	1.05 (0.96, 1.14)	199	1.07 (0.95, 1.21)	1.07 (0.95, 1.20)
0−<25%	0.01, 0.59	13982	91	1.0 (reference)	1.0 (reference)	47	1.0 (reference)	1.0 (reference)
25−<50%	0.59, 0.99	15385	100	1.03 (0.77, 1.37)	1.02 (0.77, 1.36)	40	0.80 (0.52, 1.21)	0.80 (0.53, 1.22)
50−<75%	0.99, 1.79	14850	115	1.22 (0.92, 1.60)	1.21 (0.92, 1.59)	54	1.11 (0.75, 1.63)	1.10 (0.74, 1.62)
75−<90%	1.80, 3.09	8965	61	1.12 (0.81, 1.54)	1.10 (0.80, 1.52)	33	1.17 (0.75, 1.83)	1.15 (0.73, 1.79)
≥90%	3.10, 36.55	6699	46	1.09 (0.77, 1.56)	1.08 (0.76, 1.54)	25	1.15 (0.71, 1.87)	1.16 (0.71, 1.88)
P trend^e^				0.55	0.61		0.24	0.25
**TTHM (μg/L)**	log2	59881	413	1.01 (0.98, 1.04)	1.01 (0.98, 1.04)	199	1.01 (0.97, 1.06)	1.01 (0.97, 1.06)
0−<25%	0, 0.31	14966	98	1.0 (reference)	1.0 (reference)	44	1.0 (reference)	1.0 (reference)
25−<50%	0.31, 4.55	14717	99	0.99 (0.75, 1.31)	1.00 (0.75, 1.32)	54	1.21 (0.81, 1.80)	1.22 (0.82, 1.82)
50−<75%	4.56, 20.82	15197	99	0.96 (0.73, 1.27)	0.97 (0.73, 1.28)	48	1.03 (0.69, 1.56)	1.05 (0.70, 1.58)
75−<90%	20.84, 30.86	8891	73	1.19 (0.88, 1.61)	1.19 (0.88, 1.61)	33	1.19 (0.76, 1.87)	1.21 (0.77, 1.89)
≥90%	30.94, 108.66	6110	44	1.09 (0.76, 1.55)	1.09 (0.77, 1.56)	20	1.10 (0.65, 1.86)	1.11 (0.65, 1.88)
P trend^e^				0.31	0.30		0.82	0.81

a CWS exposures[Table-fn t2fn1] were linked to the address at enrollment. Analyses were restricted to participants with a residential duration at enrollment ≥10 years^b^.

b CWS concentrations represent 15-year average (1990-2005) concentrations of arsenic (μg/L), uranium (μg/L), nitrate-N (mg/L), and TTHM (μg/L).

c Residential duration at the enrollment address was determined based on the residential history and self-reported information about duration at the current home at questionnaire 4. Mean follow-up=19.0 years, total person-years: 1,139,582. For uranium, mean follow-up=19.0 years, total person-years: 1,072,419.

d Model 1 was adjusted for baseline age (years) + baseline age^2^.

e Model 2 = model 1 + BMI category (<25 kg/m^2^, 25−<30 kg/m^2^, >30 kg/m^2^, or missing), menopause status (pre-, peri/post-menopause, or missing), live births (no/yes/missing), oral contraceptive use (never/ever/missing).

f P trend was evaluated using the median of each quantile.

**Figure 1:**
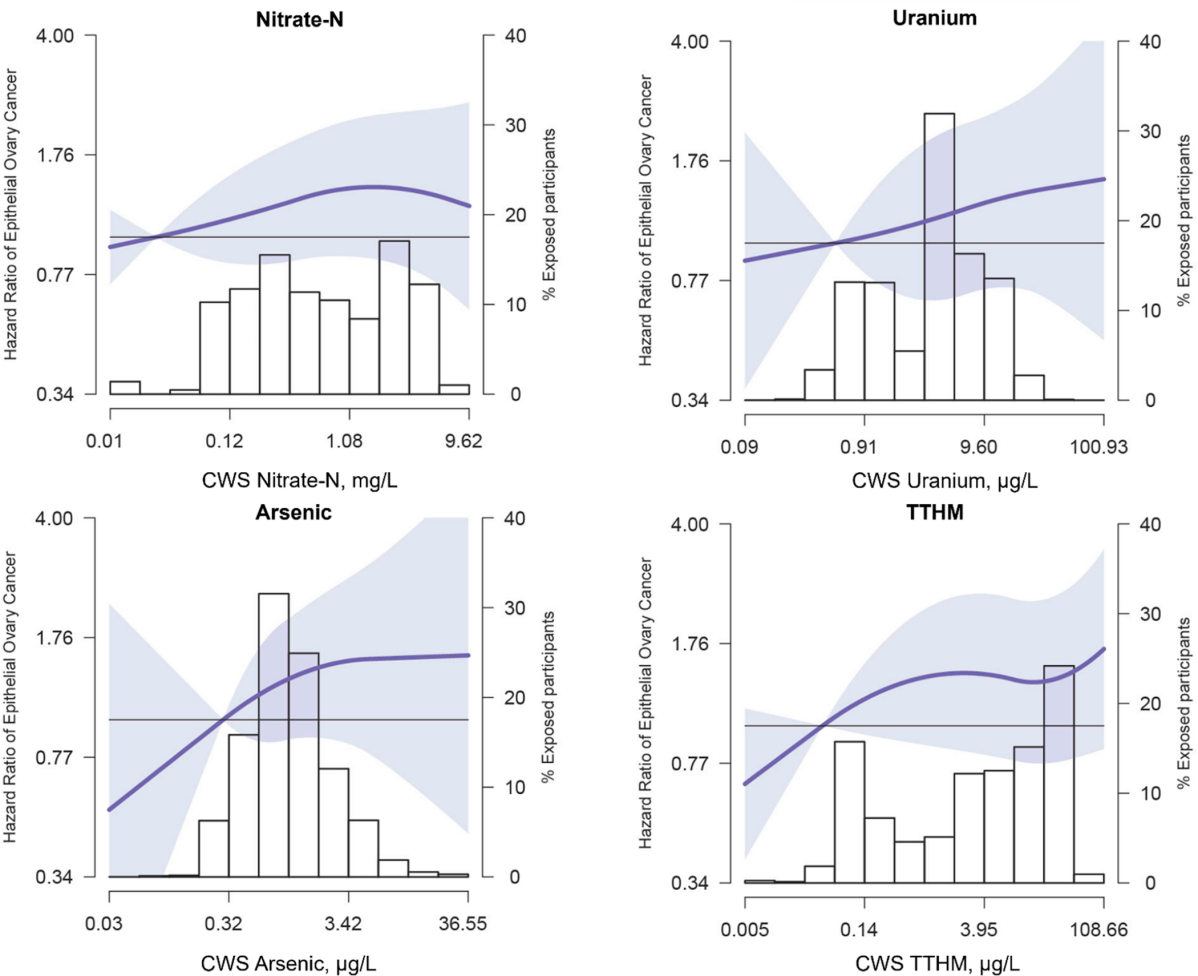
Hazard ratios (95% CI) for all epithelial ovarian carcinomas by community water system (CWS) nitrate, uranium, arsenic and total trihalomethane (TTHM) exposures in the California Teachers Study. CWS exposures^1^ were linked to the address at enrollment. Analyses were restricted to participants with a residential duration at enrollment ≥ 10 years.^2^ Lines with shaded areas represent the hazard ratios (95% confidence interval (CI)), based on cubic splines for Cox proportional hazards models using log2-transformed concentrations with knots at the 10th (reference), 50th, and 90th percentiles.^3^ A black horizontal line is at HR = 1. The histogram represents the frequency distribution of CWS estimates in the study sample.^1^ CWS concentrations represent long-term average (1990–2005) concentrations of arsenic (μg/L), uranium (μg/L), nitrate-N (mg/L), and TTHM (μg/L). For plotting purposes, several extremely low concentrations were omitted of arsenic (1 estimate = 0.008 μg/L) and TTHM (9 estimates <5.20 × 10^–3^).^2^ Residential duration at the enrollment address was determined based on the residential history and self-reported information about duration at the current home at questionnaire 4.^3^ Models were adjusted for age at baseline, age^2^, BMI category (<25 kg/m^2^, 25–<30 kg/m^2^, >30 kg/m^2^, or missing), menopause status (pre-, peri/postmenopause, or missing), live births (yes/no/missing), and oral contraceptive use (never/ever/missing).

**Figure 2:**
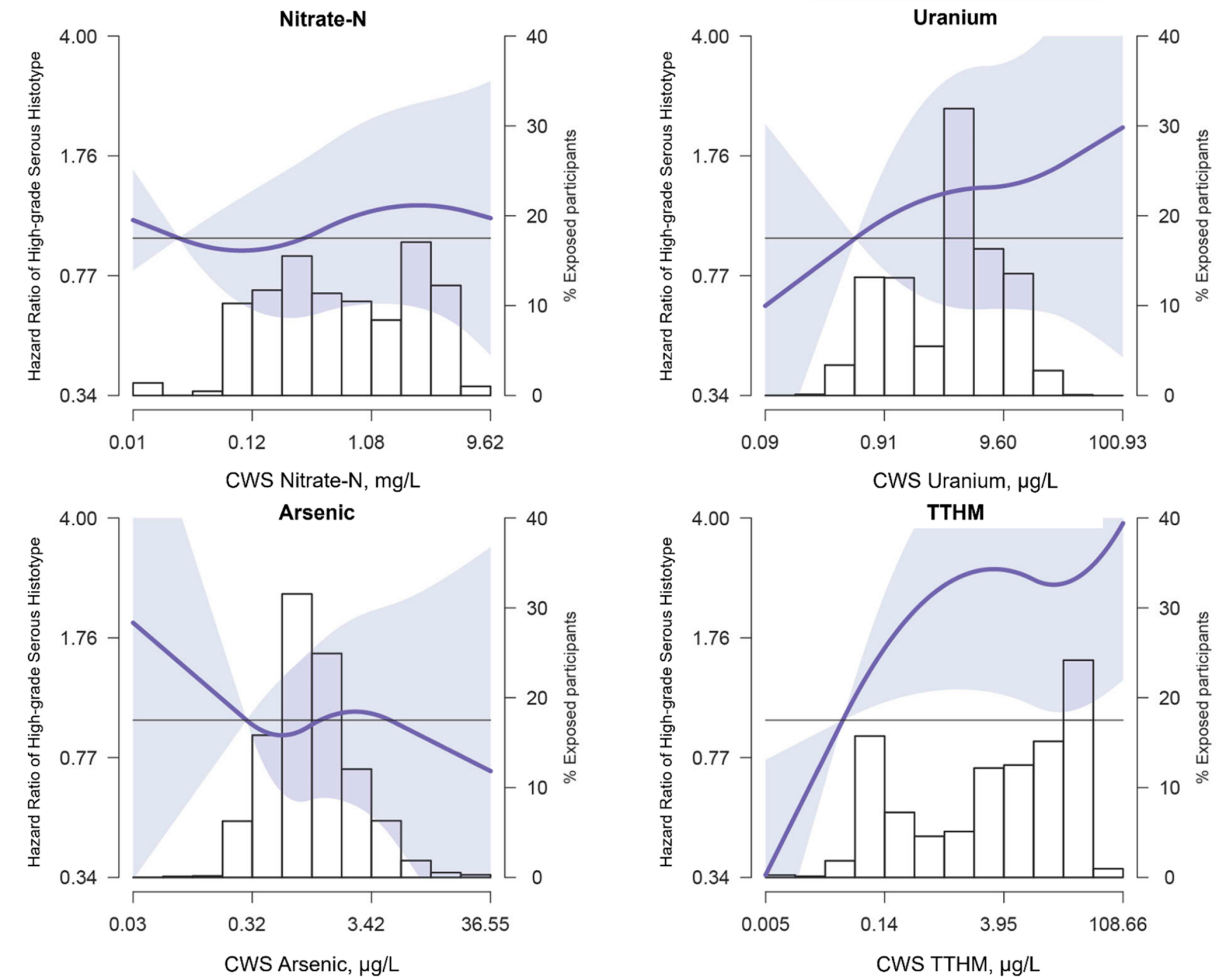
Hazard ratios (95% CI) for high-grade serous ovarian cancers by community water system (CWS) nitrate, uranium, arsenic and total trihalomethane (TTHM) exposures in the California Teachers Study. CWS exposures^1^ were linked to the address at enrollment. Analyses were restricted to participants with a residential duration at enrollment ≥ 10 years.^2^ Lines with shaded areas represent the hazard ratios (95% confidence interval (CI)), based on cubic splines for Cox proportional hazards models using log2-transformed concentrations with knots at the 10th (reference), 50th, and 90th percentiles.^3^ A black vertical line is at HR = 1. The histogram represents the frequency distribution of CWS estimates in the study sample.^1^CWS concentrations represent long-term average (1990–2005) concentrations of arsenic (μg/L), uranium (μg/L), nitrate-N (mg/L), and TTHM (μg/L). For plotting purposes, several extremely low concentrations of arsenic were omitted (1 estimate = 0.008 μg/L) and TTHM (9 estimates <5.20 × 10^–3^).^2^ Residential duration at the enrollment address was determined based on the residential history and self-reported information about duration at the current home at questionnaire 4.^3^ Models were adjusted for age at baseline, age^2^, BMI category (<25 kg/m^2^, 25–<30 kg/m^2^, >30 kg/m^2^, or missing), menopause status (pre-, peri/postmenopause, or missing), live births (yes/no/missing), and oral contraceptive use (never/ever/missing).

In analyses of joint effects, HRs per IQR increase in the contaminant mixture of all four contaminants were the largest (all ovarian cancer HR = 1.34, 95% CI 0.95, 1.88; high-grade serous cancer HR = 1.77, 95% CI 1.09, 2.87) compared to models of the contaminant mixture containing different combinations of two and three contaminants (Table 3). Uranium contributed the largest weight (56%) to the positive mixture effect on all ovarian cancers, and nitrate contributed the largest weight (48%) to the positive mixture effect on the high-grade serous histotype. In single contaminant analyses mutually adjusted for other contaminants, we observed that uranium had the largest hazard ratio for all ovarian cancer (HR = 1.18, 95% CI 0.99, 1.42), and that nitrate had the largest hazard ratio for the high-grade serous histotype (HR = 1.30, 95% CI 0.99, 1.72) (Table 3).

**Table 3:** Hazard Ratio (95% CI) for All Epithelial Ovarian Carcinomas and High Grade Serous Ovarian Cancers per Interquartile Range (IQR) Increase in the Mixture of Community Water System (CWS) Exposures and per Single Contaminant Exposures Mutually Adjusted, among California Teachers Study Participants (N=56,314)^a^

	All Epithelial Carcinomas, N Cases = 392		High-Grade Serous Histotype, N Cases = 194	
Contaminant (log2 transformed) per IQR increase	HR (95% CI) Model 1^c^	HR (95% CI) Model 2^d^	HR (95% CI) Model 1^c^	HR (95% CI) Model 2^d^
	**Single contaminants (mutually adjusted)** ^e^			
Nitrate-N	1.03 (0.85, 1.24)	1.03 (0.85, 1.24)	1.32 (1.00, 1.74)	1.30 (0.99, 1.72)
Uranium	1.19 (0.99, 1.43)	1.18 (0.99, 1.42)	0.99 (0.77, 1.28)	1.00 (0.78, 1.28)
Arsenic	0.99 (0.84, 1.17)	0.99 (0.84, 1.16)	1.07 (0.86, 1.35)	1.07 (0.85, 1.34)
TTHM	1.10 (0.90, 1.35)	1.10 (0.90, 1.35)	1.25 (0.93, 1.68)	1.25 (0.93, 1.68)
	**Mixtures models (two contaminants)** ^f^			
Nitrate-N + Uranium	1.23 (0.99, 1.52)	1.23 (0.99, 1.52)	1.32 (0.98, 1.80)	1.31 (0.97, 1.79)
Nitrate-N + Arsenic	1.03 (0.80, 1.31)	1.02 (0.80, 1.31)	1.44 (1.02, 2.02)	1.42 (1.01, 2.00)
Nitrate-N + TTHM	1.14 (0.83, 1.55)	1.14 (0.84, 1.56)	1.65 (1.05, 2.60)	1.65 (1.05, 2.59)
Uranium + Arsenic	1.18 (0.98, 1.43)	1.17 (0.97, 1.42)	1.08 (0.83, 1.41)	1.07 (0.82, 1.40)
Uranium + TTHM	1.31 (1.00, 1.72)	1.31 (0.99, 1.72)	1.24 (0.84, 1.82)	1.25 (0.85, 1.83)
Arsenic + TTHM	1.09 (0.82, 1.45)	1.09 (0.82, 1.44)	1.35 (0.91, 2.00)	1.34 (0.90, 2.00)
	**Mixtures models (three contaminants)** ^f^			
Nitrate-N + Uranium + Arsenic	1.22 (0.99, 1.51)	1.21 (0.98, 1.50)	1.43 (1.06, 1.93)	1.42 (1.05, 1.91)
Nitrate-N + Uranium + TTHM	1.35 (0.98, 1.87)	1.35 (0.98, 1.87)	1.65 (1.03, 2.63)	1.64 (1.03, 2.63)
Nitrate-N + Arsenic + TTHM	1.13 (0.79, 1.62)	1.13 (0.79, 1.62)	1.79 (1.07, 2.99)	1.77 (1.06, 2.96)
Uranium + Arsenic + TTHM	1.30 (0.97, 1.75)	1.29 (0.96, 1.74)	1.34 (0.88, 2.04)	1.34 (0.88, 2.04)
	**Mixtures models (four contaminants)** ^f^			
Nitrate-N + Uranium + Arsenic + TTHM	1.34 (0.96, 1.89)	1.34 (0.95, 1.88)	1.78 (1.10, 2.89)	1.77 (1.09, 2.87)

a CWS exposures[Table-fn t3fn1] were linked to the address at enrollment and log2 transformed. Analyses were restricted to participants with a residential duration at enrollment ≥10 years^b^.

b CWS concentrations represent 15-year average (1990-2005) concentrations of arsenic (μg/L), uranium (μg/L), nitrate-N (mg/L), and TTHM (μg/L).

c Residential duration at the enrollment address was determined based on the residential history and self-reported information about duration at the current home at questionnaire 4. Mean follow-up = 19.0 years, total person-years: 1,072,419.

d Model 1 was adjusted for baseline age (years) + baseline age^2^.

e Model 2 = model 1 + BMI category (<25 kg/m^2^, 25−<30 kg/m^2^, >30 kg/m^2^, or missing), menopause status (pre-, peri/post-menopause, or missing), live births (no/yes/missing), oral contraceptive use (never/ever/missing).

f Concentrations were log2 transformed and subsequently divided by the IQR. Models were adjusted for all other contaminants (log2 transformed).

g For contaminants included in the mixture, concentrations were log2 transformed and subsequently divided by the IQR. The effect of the drinking water contaminant mixture was evaluated using quantile q-computation, and hazard ratios are interpreted per IQR increase. Models were adjusted for contaminants not in the mixture (log2 transformed).

Compared to participants who had no years of exposure to nitrate ≥ 1/2 MCL (0%), having >10% years of exposure to nitrate was associated with an elevated risk of high-grade serous histotype (HR= 1.72, 95% CI 1.20, 2.46), whereas no association was observed with all ovarian cancer (Table 4). Having >10% of years of exposure to uranium ≥ 1/2 MCL versus 0% was positively associated with all ovarian cancer (HR= 1.22, 95% CI 0.94, 1.57) and the high-grade serous histotype (HR= 1.28, 95% CI 0.90, 1.83). We observed suggestive positive associations for >0% of years of exposure to arsenic ≥ 1/2 MCL and the high-grade serous histotype, and no associations for TTHM.

**Table 4:** Hazard Ratios (95% CI) for All Epithelial Ovarian Carcinomas and High Grade Serous Ovarian Cancers by the Percentage of Years of Community Water System (CWS) Exposures Exceeding 1/2 of the Maximum Contaminant Levels (MCLs)^a^ (1990-2005), among California Teachers Study Participants^a^

		All Epithelial Carcinomas			High-grade Serous Histotype		
% years > MCL/2	N	N cases	HR (95% CI) Model 1^c^	HR (95% CI) Model 2^d^	N Cases	HR (95% CI) Model 1^c^	HR (95% CI) Model 2^d^
**Nitrate-N**							
0% years	47696	325	1.0 (ref)	1.0 (ref)	142	1.0 (ref)	1.0 (ref)
>0−≤10% years	4574	34	1.09 (0.77, 1.56)	1.08 (0.76, 1.54)	19	1.40 (0.87, 2.26)	1.40 (0.87, 2.26)
>10% years	7611	54	1.08 (0.81, 1.44)	1.07 (0.81, 1.43)	38	1.74 (1.21, 2.49)	1.72 (1.20, 2.46)
**Uranium**							
0% years	44619	300	1.0 (ref)	1.0 (ref)	148	1.0 (ref)	1.0 (ref)
>0−≤10% years	2540	17	1.04 (0.64, 1.69)	1.03 (0.63, 1.68)	7	0.86 (0.40, 1.84)	0.87 (0.41, 1.85)
>10% years	9155	75	1.23 (0.95, 1.58)	1.22 (0.94, 1.57)	39	1.29 (0.91, 1.84)	1.28 (0.90, 1.83)
**Arsenic**							
0% years	41274	276	1.0 (ref)	1.0 (ref)	128	1.0 (ref)	1.0 (ref)
>0−≤10% years	10717	84	1.17 (0.92, 1.50)	1.17 (0.92, 1.49)	42	1.26 (0.89, 1.79)	1.25 (0.88, 1.77)
>10% years	7890	53	1.04 (0.78, 1.40)	1.03 (0.77, 1.38)	29	1.23 (0.82, 1.84)	1.21 (0.81, 1.82)
**TTHM**							
0% years	36689	249	1.0 (ref)	1.0 (ref)	121	1.0 (ref)	1.0 (ref)
>0−≤10% years	5978	36	0.86 (0.60, 1.22)	0.87 (0.61, 1.23)	18	0.88 (0.54, 1.45)	0.89 (0.54, 1.45)
>10% years	17214	128	1.07 (0.86, 1.32)	1.07 (0.86, 1.32)	60	1.03 (0.75, 1.4)	1.03 (0.76, 1.41)

a CWS exposures were linked to the address at enrollment. Analyses were restricted to participants with a residential duration at enrollment ≥10 years^b^.

b The percent (%) of years was calculated as the number of years that average annual concentrations exceeded 1/2 the MCL / the number of years of monitoring data reported for each contaminant per CWS, and modeled as categorical variables comparing 0% (reference), >0−≤10%, and >10% of years. One half of the MCLs are as follows: arsenic (5 μg/L), uranium (15 μg/L), gross alpha (7.5 pCi/L, not including radon and uranium), nitrate-N (5 mg/L), TTHM (40 μg/L).

c Residential duration at the enrollment address was determined based on the residential history and self-reported information about duration at the current home at questionnaire 4. Mean follow-up=19.0 years, total person-years: 1,139,582. For uranium, mean follow-up=19.0 years, total person-years: 1,072,419.

d Model 1 was adjusted for baseline age (years) + baseline age^2^.

e Model 2 = model 1 + BMI category (<25 kg/m^2^, 25-<30 kg/m^2^, >30 kg/m^2^, or missing), menopause status (pre-, peri/post-menopause, or missing), live births (no/yes/missing), oral contraceptive use (never/ever/missing).

In time varying analyses of cumulative average exposures lagged 5 years (Table S3), we observed a positive association per doubling in nitrate concentrations for the high-grade serous histotype (HR = 1.06, 95% CI 1.00, 1.12) and positive associations by quantile of exposure (p-trend = 0.09). We observed a positive association with all ovarian cancer for the 90th percentile of uranium exposure (HR = 1.15, 95% CI 0.84, 1.56; p-trend = 0.42) and for the high-grade serous histotype with a doubling in arsenic concentrations (HR = 1.06, 95% CI 0.97, 1.16). Findings were generally similar compared to the main analyses, although associations with nitrate, uranium, and arsenic were attenuated; as with the main analyses, there were no associations with TTHM. In posthoc analyses limited to the same residentially stable population as the main analyses, the magnitude of associations with time-varying exposures were more consistent with results from the main analyses (Table S4).

We noted only minor differences in the associations between a doubling in CWS exposures and ovarian cancer risk by menopausal status and BMI category (Table S5). We observed no evidence of statistical interaction when we stratified by pre- and peri/postmenopausal status, though the HRs for arsenic were higher among premenopausal women. Arsenic was positively associated with ovarian cancer among participants with a BMI 25–<30 kg/m^2^ (HR = 1.28, 95% CI 1.10, 1.49) but not among those with lower or higher BMI. We did not observe evidence of statistical interaction by smoking status (Table S6).

Median average concentrations of nitrate, uranium, and arsenic were higher among participants living in nonmetropolitan areas; whereas median average TTHM concentrations were higher among participants in metropolitan areas. Nitrate, uranium, and arsenic were moderately positively correlated with each other, and negatively correlated with TTHM, in both nonmetropolitan and metropolitan areas (Figure S4). In single contaminant and mixture analyses, we observed stronger associations in the risk of all ovarian and high-grade serous histotype cancers per increase in nitrate, uranium, and arsenic among nonmetropolitan participants compared to metropolitan participants, although there was no evidence of statistical interaction (p-interaction ≥ 0.05) in fully adjusted analyses (Table S7). In analyses stratified by CWS size, we observed stronger HRs for all ovarian and high-grade serous cancers per doubling in nitrate, uranium, and arsenic among medium-sized CWS compared with large and very large CWS. There was no evidence for statistical interaction by CWS size, except for nitrate for all ovarian cancer risk (p-interaction = 0.05; Table S8). Associations with ovarian cancer risk were similar across groundwater and surface water sources, and we did not observe evidence for statistical interaction by water source type (*p* ≥ 0.05; Table S9).

Associations with nitrate, uranium, and arsenic were stronger among participants in the lower two tertiles of vitamin C intake compared to those in the highest tertile (p-interactions > 0.05). HRs for high-grade serous cancer were also higher in the lower two tertiles of vitamin C intake but there were no statistical interactions (p interactions ≥ 0.05; Table S10-B). Though we did not observe a statistical interaction between tertiles of red meat intake and CWS nitrate, we observed somewhat stronger associations between nitrate and ovarian cancer among participants in the second tertile of total daily red meat intake (19.0–41.1 g/day; Table S11) compared to the other tertiles. Further, we observed increased risk of high-grade serous cancer at higher quartiles of CWS nitrate among participants in the second tertile (p trend <0.05) of red meat intake.

In analyses of dietary intakes of nitrate and nitrite (Table S12), we did not observe significant associations with all ovarian cancers or the high-grade serous histotype for total dietary nitrate, total dietary nitrite, or for nitrite intake from plant or processed meat sources. However, we observed increasing risk of all ovarian cancer with increasing quartiles of dietary nitrite from animal sources (p trend <0.05).

We observed positive associations per doubling of gross alpha levels for all ovarian cancer (HR = 1.11, 95% CI 1.01, 1.22) (Table S13) that were generally similar to the results for uranium. For regulatory purposes, gross alpha radioactivity is measured as the sum of α particle activity, which may be released from uranium and other radionuclides. As many CWS in California (particularly smaller systems) did not provide uranium measurements when gross alpha levels were <5 pCi/L, our findings suggest that gross alpha levels, which were highly correlated with uranium, may be a useful proxy for uranium exposure in epidemiologic analyses in California.

In sensitivity analyses in which we started follow-up on January 1, 2005, we observed results similar to those of our main single contaminant and four contaminant mixture findings (Table S14; Table S13 for gross alpha). We separately restricted our main single contaminant and four contaminant mixture analyses to participants whose 15 year average contaminant exposures were below the corresponding MCLs; these findings were consistent with the main analyses (Table S15).

## 4 Discussion

In this large prospective cohort of women in California, we evaluated exposures to frequently detected and regulated contaminants in public water supplies and ovarian cancer risk. We observed positive associations between uranium and all epithelial ovarian cancers and between nitrate and the high-grade serous histotype. We observed the strongest positive associations per IQR increase in the mixture that included nitrate, uranium, arsenic, and TTHM. The joint effect generally increased as contaminants were added to the mixture model, suggesting that single contaminants (i.e., arsenic and TTHM) that did not have a statistically significant effect individually may nonetheless contribute to an overall drinking water mixture effect on ovarian cancer risk.

Few previous cohort studies have evaluated the association between drinking water contaminants and epithelial ovarian cancer risk. The Iowa Women’s Health Study, a prospective cohort of postmenopausal women, estimated long-term average nitrate and disinfection byproduct (TTHM and haloacetic acids) exposures in CWS based on participants’ enrollment address.^11^ They found a significant elevated risk of ovarian cancer with increasing quartiles of average CWS nitrate exposure (highest quartile ≥ 2.98 mg/L) in models adjusted for TTHM.^11^ TTHM and haloacetic acid exposures were not associated with ovarian cancer risk, and there was no evidence for interaction with nitrate. We observed an elevated risk of high-grade serous ovarian cancer among participants who had >10% of years of exposure to nitrate ≥ 5 mg/L (1.68, 95% CI 1,17, 2.39); this is consistent with the evaluation in the Iowa Women’s Health Study, which yielded an elevated risk for ovarian cancer participants who had ingested water with nitrate levels >5 mg/L for at least 4 years compared to those with no years of exposure at this level (HR = 1.60, 95% CI 1.06, 2.41).^11^ Similarly, nitrate was positively associated with ovarian cancer risk in the Agricultural Health Study (HR_per 5 mg/L_ = 1.15, 95% CI 0.96, 1.39), a prospective cohort of pesticide applicators and their spouses primarily using private wells for their drinking water in rural areas of Iowa and North Carolina.^12^

Studies of carcinogenic effects of NOCs in multiple animal species provide biologic plausibility for an epidemiologic association between drinking water nitrate and ovarian cancer risk.^13,58,59^ Prior epidemiologic evidence for other drinking water contaminants and ovarian cancer risk are limited. To the best of our knowledge, no prior studies evaluated uranium in drinking water and ovarian cancer risk. Uranium is a potent toxicant associated with reproductive toxicity in animal and mechanistic studies, providing biological plausibility for the novel epidemiologic associations we observed for uranium.^16^ Animal studies showed that uranium ingested from drinking water accumulates in the ovaries and causes DNA hypomethylation.^16^ In cell-based studies, uranium was associated with decreased germ cell density and an increased apoptosis rate in human fetal ovaries.^60^

A study of ovarian cancer mortality rates in an area of Chile that experienced large fluctuations in arsenic levels in drinking water found a reduction in ovarian cancer mortality during a period of high exposure (mean levels of 870 μg/L) compared to earlier and later periods with lower arsenic levels (<100 ug/L).^61^ Animal studies indicate that transplacental exposure to inorganic arsenic can induce ovarian tumors in the offspring of mice, so timing of exposures may be important.^17^ A hospital-based case-control study of women with primary ovarian insufficiency in China found that urinary arsenic levels were higher among cases compared to healthy controls matched by age and BMI.^62^ Primary ovarian insufficiency may be linked to an increased risk of ovarian cancer; evidence is limited.^63^ Arsenic is a known carcinogen associated with reproductive toxicity in human, animal and mechanistic studies, however future studies with historical measurements and a greater range in exposure are needed to determine whether arsenic is associated with ovarian cancer risk.^17,19,64–66^

We observed non-statistically significant effect modification of the associations of water uranium and arsenic stratified by vitamin C intake; specifically, the risk of all ovarian cancer was attenuated among participants in the highest tertile of vitamin C intake. Limited evidence suggests that vitamin C intake may decrease metal/metalloid toxicity through multiple potential mechanistic pathways (e.g., oxidative stress) and through enhancing metal excretion in urine.^57^ Similar to our findings in the CTS, stronger associations were observed between water nitrate and ovarian cancer at lower levels of vitamin C intake (<median, 190 mg/day) in the Iowa Women’s Health Study.^11^ A dietary pattern of high intake of nitrate from drinking water and low intake of vitamin C increases endogenous formation of NOCs.^13^ Diet can be an important source of exposure to arsenic (e.g., rice) and uranium (e.g., root crops). In the CTS, rice intake was previously associated with a modest increased risk of breast cancer, but not with lung, pancreatic, bladder, or kidney cancers; ovarian cancer risk was not evaluated.^67^ As arsenic and uranium levels in diet and biomarker data were not measured in this cohort, we could not evaluate associations with dietary arsenic or uranium; this represents an area of future research.

We observed an interaction between water arsenic and BMI on all ovarian cancer risk (p interaction < 0.05), however the pattern was not clear (i.e., elevated risk was only observed in the middle [overweight] BMI category). We also found that the risk of ovarian cancer per doubling in water arsenic was higher among premenopausal women, however the interaction was not statistically significant. Prior evidence suggests that the effect of adiposity on ovarian cancer risk may vary by hormone therapy use, menopausal status, and histotype.^44,68,69^ Further investigation is needed to understand the potential interaction of metals/metalloids with body weight and composition in the association between water metals/metalloids and ovarian cancer.

Strengths of our study included the nearly 60,000 women in the CTS with >10 years on their enrollment residence CWS, and comprehensive follow-up and information on demographic, anthropometric, dietary, and medical factors, including annual linkages to California cancer and mortality registries. We estimated 15-year average drinking water exposures using robust water quality monitoring data and CWS distribution boundaries, which have been extensively described and were previously validated for use in this cohort using self-reported drinking water information at a later follow-up (Q6).^32^

Our study also had some limitations. The lack of historical data before 1990 limited our ability to assess early life and long-term exposures in this study. We partly addressed this by limiting our analyses to women who lived a minimum of 10 years at their enrollment address. This approach assumes that the 15-year average reflects earlier exposures, which may have led to misclassification of exposure if the concentrations were different prior to 1990. We expect that a temporal overlap of the exposure (1990–2005) and follow-up (beginning in 1995) periods did not bias our findings, as we observed similar results in sensitivity analyses that started follow-up in 2005. In time-varying analyses, we incorporated residential changes over time and computed a cumulative average exposure estimate that was lagged 5 years. A 5-year lag was the maximum we could apply without extrapolating to time periods where data were not available; however, a 5-year lag is likely insufficient to capture the latency period for ovarian cancer.^70^ Residence duration was asked in the fourth, fifth, and sixth questionnaires. For other questionnaire addresses and addresses assigned by linkages, the move-in date was estimated as the minimum date among matched records, which may have led to misclassification of exposure.^35^ However, in analyses that evaluated time-varying exposures in the same residentially stable population as the main analysis, our findings were more similar to the results observed from using the 15-year average drinking water exposure metric. Overall, we observed consistent patterns across different drinking water exposure metrics.

Additional limitations include our exposure assessment based on residential address rather than self-reported drinking water source. Drinking water source and tap water treatment were only collected at the sixth follow-up questionnaire (Q6, 2017–2019; response rate 43%); therefore, we were not able to use this information in our exposure assessment. However, among participants who provided this information at Q6 (*N* = 33,276), we found that self-reported and geocoded address-assigned water source had high agreement,^32^ indicating that exposure misclassification of the water source is not likely to be large. Specifically, among participants linked to a CWS based on their Q6 address, 74% reported their tap water source as municipal water, 2% as private well water, 15% as bottled water, 4% as other, and 5% as do not know/missing.^32^ Participants who drank only bottled water may still have exposure to inorganic contaminants via ingestion from tap water used for cooking and consumption of beverages made with boiling water and to THMs through inhalation and dermal exposure.

Finally, differences in water quality by sociodemographic characteristics (e.g., by race and ethnicity, neighborhood urbanicity and SES) have been observed in the CTS and across California.^32,71^ The exposure distributions in our study population (an educated cohort of women who predominantly lived in metropolitan (including suburban) areas at enrollment and are predominantly non-Hispanic white) may not reflect the full range of exposures to California women. This underscores the need to replicate these analyses in additional study populations to advance our understanding of ovarian cancer etiology.

## Conclusions

We observed positive associations between CWS uranium and nitrate levels and the risk of all epithelial and high-grade serous ovarian cancer, respectively. We also found a positive effect of the drinking water contaminant mixture on all ovarian and high-grade serous cancer. Although future research is needed to provide additional epidemiologic support for these novel findings, our results suggest that uranium exposure through drinking water may be a novel risk factor for epithelial ovarian carcinomas and that nitrate in drinking water is a risk factor for high-grade serous cancer. The identification of modifiable risk factors for high-grade serous cancer is particularly important, as it has a poor prognosis compared to other histotypes. There is a critical need for more epidemiological studies of gynecologic cancers and drinking water contaminant exposures. If these findings are confirmed, population-level interventions to reduce drinking water nitrate and uranium levels may be potential opportunities to reduce ovarian cancer risk from environmental exposures.

## Supplementary Material


