# Supplementary material for: Exposures to Drinking Water Contaminants in Community Water Systems and Risk of Ovarian Cancer in the California Teachers Study Cohort
Source: Environ Health Perspect. 2026 May 20;134(4):452–64. doi: 10.1021/EHP.6c00169 (PMC13445266; doi:10.1021/EHP.6c00169)
Supplement: Supplementary file 1 [file hp6c00169_si_001.pdf]

Supplemental Tables and Figures

Page (s)	Table	Title
2	S1	Detection limits for the purposes of reporting (DLR), and upper bound concentrations used for imputing concentrations <DLR.
3	S2	Compliance cycles and monitoring periods for inorganic, radionuclide, and disinfection byproduct contaminants (DBP) in community water systems (CWS) regulated by the U.S. Environmental Protection Agency.
4-5	S3	Hazard ratios (HRs) (95% CI) for all epithelial ovarian carcinomas and high grade serous ovarian cancers per doubling and quantiles of time-varying CWS exposures among California Teachers Study (CTS) participants.
6-7	S4	HRs (95% CI) for all epithelial ovarian carcinomas and high grade serous ovarian cancers per doubling and quantiles of time-varying CWS exposures, among CTS participants who were included in the main analysis (CWS linked to the enrollment address, residential duration at enrollment ≥ 10 years).
8-9	S5	HRs (95% CI) for all epithelial ovarian carcinomas and high grade serous ovarian cancers per doubling of CWS exposures among CTS participants, by menopause and body mass index (BMI) category.
10	S6	HRs (95% CI) for all epithelial ovarian carcinomas and high grade serous ovarian cancers per doubling of CWS exposures among CTS participants, by smoking status.
11-12	S7	HRs (95% CI) for all epithelial ovarian carcinomas and high grade serous ovarian cancers per doubling of CWS exposures, and per interquartile range increase in the contaminant mixture, among CTS participants stratified by urbanicity.
13-14	S8	HRs (95% CI) for all epithelial ovarian carcinomas and high grade serous ovarian cancers per doubling of CWS exposures, among CTS participants stratified by the size of the CWS population served.
15	S9	HRs (95% CI) for all epithelial ovarian carcinomas and high grade serous ovarian cancers per doubling of CWS exposures, and per interquartile range increase in the contaminant mixture, among CTS participants stratified by CWS water source.
16-17	S10A-B	HRs (95% CI) for all epithelial and high-grade serous ovarian carcinomas per doubling and quartile of CWS exposures among CTS participants, stratified by tertile of by vitamin C intake (mg/day).
18	S11	HRs (95% CI) for all epithelial ovarian carcinomas and high grade serous ovarian cancers per doubling and quartile of CWS exposures among CTS participants, stratified by tertile of by total red meat intake (g/day).
19-20	S12	HRs (95% CI) for all epithelial ovarian carcinomas and high grade serous ovarian cancers per doubling and quartile (Q) of dietary nitrate and nitrite intake (mg/day) among CTS participants.
21-22	S13	HRs (95% CI) for all epithelial ovarian carcinomas and high grade serous ovarian cancers per doubling and categories of CWS gross alpha exposures, among CTS participants.
23-24	S14	HRs (95% CI) for all epithelial ovarian carcinomas and high grade serous ovarian cancers per doubling of CWS exposures, and per interquartile range increase in the contaminant mixture, among CTS participants, excluding participants censored prior to 2005.
25	S15	HRs (95% CI) for all epithelial ovarian carcinomas and high grade serous ovarian cancers per doubling of CWS exposures, and per interquartile range increase in the contaminant mixture, among CTS participants with exposures < maximum contaminant levels (MCL).
**Page (s)**	**Figure**	**Title**
26	S1	Inclusion and exclusion criteria of CWS exposures and ovarian cancer risk in the CTS.
27	S2	Map of CWS serving CTS participants in California.
28	S3	Spearman correlation coefficients of 15-year average (1990-2005) CWS arsenic, uranium, gross alpha, nitrate-nitrogen (N), and total trihalomethanes (TTHM) concentrations among CTS participants.
29	S4	Spearman correlation coefficients of 15-year average (1990-2005) CWS arsenic, uranium, gross alpha, nitrate-nitrogen (N), and total trihalomethanes (TTHM) concentrations among CTS participants, among non-metropolitan *(left)* and metropolitan participants *(right)*.

Table S1. Detection limits for the purposes of reporting (DLR), and upper bound concentrations used for imputing concentrations <DLR[1].

Contaminant	Unit	DLR	Upper bound
Nitrate as N	mg/L	0.4	0.1
Arsenic	µg/L	2	1 (prior to 2008);<br>0.5 (2008 onwards)
Uranium	pCi/L	1	0.5
Gross Alpha	pCi/L	3	1
Total Trihalomethanes (TTHM)	µg/L	0.0<br>(None)	0.5

[1]When samples had a concentration of 0 or were reported as "below the DLR", we imputed concentrations using Tobit regression based on a log-normal distribution for the existing measurement data. The upper bound for imputation was derived from the median of reported concentrations below the DLR and the lower bound was zero.

Table S2. Compliance cycles and monitoring periods for inorganic, radionuclide, and disinfection byproduct contaminants (DBP) in community water systems (CWS) regulated by the U.S. Environmental Protection Agency. For years that CWS were missing annual monitoring data we interpolated data using the average concentration from the corresponding monitoring period, and if unavailable, we substituted the average estimate from the 1) earlier monitoring period or, if unavailable, 2) later monitoring period, within the corresponding compliance cycle. For the inorganics, each compliance cycle (1: 1993-2001; 2:2002-2010; 3: 2011-2019) has a different shade of blue. For the radionuclides, we defined compliance cycles as 1990-2007 and 2008-2019 (colored in two shades of blue). Of note, we included data from 1990-1992 in the 1st compliance cycle, and data from 2020 in the most recent monitoring period for all contaminants (shown below). For the interpolation of missing data for the DBPs, we grouped annual monitoring data into two periods: 1990-2013, and 2014-2020 (locational annual average monitoring was in effect for all systems by October 1, 2013), as colored in two shades of blue.[1-6] For years that CWS were missing DBP, we interpolated data using the average concentration from the corresponding monitoring period, and if unavailable, we substituted the average estimate from 1990-2020.

**Compliance cycle**	**1st cycle (1993-2001)**				**2nd cycle (2002-2010)**			**3rd cycle (2011-2019)**			
**Monitoring period**	**1990-1992**	**1993-1995**	**1996-1998**	**1999-2001**	**2002-2004**	**2005-2007**	**2008-2010**	**2011-2013**	**2014-2016**	**2017-2019**	**2020**
**Arsenic, Nitrate-N (Inorganics)**						Initial monitoring for Final Arsenic Rule (FAR)	Compliance monitoring for FAR begins				
**Uranium, Gross Alpha (Radionuclides)**					2000-2003 Grandfathered Data collected	2004-2007 Initial monitoring	Compliance monitoring begins				
**Total trihalomethanes (Disinfection Byproducts)**									Locational running annual average (LRAA) monitoring begins		

Table S3. Hazard ratios (95% CI) for all epithelial ovarian carcinomas and high grade serous ovarian cancers per doubling and quantiles of time-varying, community water system (CWS) exposures among California Teachers Study participants. CWS exposures were linked to address(es) from the residential history and represent cumulative average exposures of arsenic (µg/L), uranium (µg/L), nitrate-N (mg/L), and total trihalomethanes (TTHM, µg/L) lagged 5 years.

		All Epithelial Carcinomas			High-grade Serous Histotype		
	CWS concentration	N cases	HR (95% CI) Model 1[1]	HR (95% CI) Model 2[2]	N Cases	HR (95% CI) Model 1[1]	HR (95% CI) Model 2[2]
**Nitrate-N**, N=85030, total person-years=1,550,260		564			281		
Log2			1.01 (0.97, 1.05)	1.01 (0.97, 1.05)		1.06 (1.00, 1.12)	1.06 (1.00, 1.12)
0-<25%	0.01, 0.20	136	1.0 (reference)	1.0 (reference)	63	1.0 (reference)	1.0 (reference)
25-<50%	0.20, 0.57	146	1.02 (0.81, 1.30)	1.02 (0.81, 1.29)	62	0.93 (0.66, 1.33)	0.93 (0.65, 1.32)
50-<75%	0.57, 2.10	141	1.04 (0.82, 1.32)	1.04 (0.82, 1.32)	77	1.22 (0.87, 1.70)	1.22 (0.87, 1.70)
75-<90%	2.10, 3.78	88	1.08 (0.83, 1.41)	1.08 (0.82, 1.41)	49	1.29 (0.89, 1.88)	1.29 (0.89, 1.88)
>=90%	3.78, 19.22	53	0.99 (0.72, 1.37)	0.99 (0.72, 1.37)	30	1.22 (0.79, 1.88)	1.21 (0.78, 1.87)
P trend[3]			0.90	0.90		0.13	0.13
**Uranium**, N=78101, total person-years=1,417,478		531			269		
Log2			1.02 (0.96, 1.08)	1.02 (0.96, 1.08)		0.97 (0.90, 1.06)	0.97 (0.90, 1.05)
0-<25%	0.09, 1.84	132	1.0 (reference)	1.0 (reference)	73	1.0 (reference)	1.0 (reference)
25-<50%	1.84, 4.46	128	1.05 (0.82, 1.34)	1.05 (0.82, 1.34)	63	0.94 (0.67, 1.31)	0.94 (0.67, 1.31)
50-<75%	4.46, 6.48	135	1.07 (0.84, 1.37)	1.07 (0.84, 1.36)	73	1.06 (0.76, 1.46)	1.06 (0.76, 1.46)
75-<90%	6.48, 10.55	78	1.04 (0.79, 1.38)	1.04 (0.79, 1.38)	37	0.89 (0.60, 1.32)	0.89 (0.60, 1.32)
>=90%	10.55, 101.62	58	1.16 (0.85, 1.57)	1.15 (0.84, 1.56)	23	0.84 (0.52, 1.34)	0.84 (0.52, 1.34)
P trend[3]			0.4	0.42		0.46	0.45
**Arsenic**, N=84899, total person-years=1,547,871		564			281		
Log2			1.03 (0.97, 1.10)	1.03 (0.97, 1.10)		1.06 (0.97, 1.17)	1.06 (0.97, 1.16)
0-<25%	0, 0.56	134	1.0 (reference)	1.0 (reference)	66	1.0 (reference)	1.0 (reference)
25-<50%	0.56, 1.01	140	1.04 (0.82, 1.32)	1.04 (0.82, 1.32)	60	0.90 (0.63, 1.28)	0.90 (0.63, 1.28)
50-<75%	1.01, 1.80	150	1.15 (0.91, 1.45)	1.14 (0.90, 1.45)	78	1.22 (0.87, 1.69)	1.21 (0.87, 1.69)
75-<90%	1.80, 3.31	87	1.13 (0.86, 1.49)	1.13 (0.86, 1.48)	49	1.29 (0.89, 1.87)	1.28 (0.88, 1.86)
>=90%	3.31, 100	53	1.05 (0.76, 1.44)	1.05 (0.76, 1.44)	28	1.13 (0.72, 1.75)	1.13 (0.72, 1.76)
P trend[3]			0.69	0.69		0.26	0.26
**TTHM**, N=86241, total person-years=1,573,839		569			285		
Log2			1.01 (0.98, 1.04)	1.01 (0.98, 1.04)		1.01 (0.96, 1.05)	1.01 (0.96, 1.05)

0-<25%	0, 1.36	143	1.0 (reference)	1.0 (reference)	73	1.0 (reference)	1.0 (reference)
25-<50%	1.36, 7.96	125	0.83 (0.65, 1.06)	0.83 (0.65, 1.06)	59	0.76 (0.54, 1.08)	0.77 (0.55, 1.09)
50-<75%	7.96, 19.80	138	0.92 (0.72, 1.16)	0.92 (0.72, 1.16)	67	0.86 (0.62, 1.20)	0.87 (0.62, 1.21)
75-<90%	19.80, 29.84	94	1.00 (0.77, 1.30)	1.00 (0.77, 1.30)	48	1.00 (0.69, 1.44)	1.00 (0.69, 1.44)
>=90%	29.84, 172.05	69	1.13 (0.85, 1.51)	1.13 (0.85, 1.51)	38	1.22 (0.82, 1.80)	1.21 (0.82, 1.80)
P trend[3]			0.18	0.18		0.14	0.16

[1]Model 1 was adjusted for age (time-varying) + age^2.
[2]Model 2 = model 1 + BMI category (<25 kg/m^2, 25-<30 kg/m^2, >30 kg/m^2, or missing), menopause status (pre-, peri/post-menopause, or missing), live births (yes/no/missing), oral contraceptive use (never/ever/missing).
[3]P trend was evaluated using the median of each quantile.

Table S4. Hazard ratios (95% CI) for all epithelial ovarian carcinomas and high grade serous ovarian cancers per doubling and quantiles of time-varying, community water system (CWS) exposures, among California Teachers Study participants. CWS exposures were linked to address(es) from the residential history and represent cumulative average exposures of arsenic (µg/L), uranium (µg/L), nitrate-N (mg/L), and total trihalomethanes (TTHM, µg/L) lagged 5 years. Analyses were restricted to participants who were included in the main analysis (CWS linked to the enrollment address, residential duration at enrollment ≥ 10 years).

		All Epithelial Carcinomas			High-grade Serous Histotype		
	**CWS concentration**	**N cases**	**HR (95% CI) Model 1[1]**	**HR (95% CI) Model 2[2]**	**N Cases**	**HR (95% CI) Model 1[1]**	**HR (95% CI) Model 2[2]**
**Nitrate-N**, N=57786, total person-years=1097807							
Log2		402	1.03 (0.98, 1.08)	1.02 (0.98, 1.07)	192	1.10 (1.02, 1.18)	1.10 (1.02, 1.18)
0-<25%	(0.01,0.19)	93	1.0 (reference)	1.0 (reference)	38	1.0 (reference)	1.0 (reference)
25-<50%	(0.19,0.61)	98	1.00 (0.75, 1.33)	1.00 (0.75, 1.33)	40	0.99 (0.64, 1.55)	0.99 (0.64, 1.55)
50-<75%	(0.61,2.19)	112	1.19 (0.90, 1.57)	1.19 (0.90, 1.57)	54	1.40 (0.92, 2.12)	1.39 (0.91, 2.10)
75-<90%	(2.19,3.90)	63	1.11 (0.80, 1.53)	1.10 (0.80, 1.52)	38	1.63 (1.04, 2.56)	1.61 (1.03, 2.53)
>=90%	(3.90,19.22)	36	0.99 (0.67, 1.45)	0.98 (0.67, 1.45)	22	1.47 (0.87, 2.49)	1.45 (0.86, 2.45)
P trend[3]			0.86	0.89		0.03	0.03
**Uranium**, N=53034, total person-years=1005466							
Log2		379	1.06 (0.99, 1.13)	1.06 (0.99, 1.13)	185	1.01 (0.92, 1.11)	1.01 (0.92, 1.11)
0-<25%	(0.09,1.48)	80	1.0 (reference)	1.0 (reference)	44	1.0 (reference)	1.0 (reference)
25-<50%	(1.48,4.40)	99	1.31 (0.98, 1.76)	1.31 (0.97, 1.76)	50	1.20 (0.80, 1.81)	1.20 (0.80, 1.80)
50-<75%	(4.40,6.54)	97	1.27 (0.94, 1.71)	1.26 (0.94, 1.7)	44	1.05 (0.69, 1.59)	1.04 (0.68, 1.58)
75-<90%	(6.54,10.57)	64	1.40 (1.01, 1.95)	1.40 (1.01, 1.95)	30	1.18 (0.74, 1.87)	1.17 (0.73, 1.86)
>=90%	(10.57,101.62)	39	1.25 (0.85, 1.84)	1.23 (0.84, 1.81)	17	0.99 (0.57, 1.73)	0.98 (0.56, 1.72)
P trend[3]			0.18	0.20		0.99	0.97
**Arsenic**, N= 57709, total person-years=1096315							
Log2		402	1.06 (0.98, 1.14)	1.05 (0.98, 1.14)	192	1.11 (1.00, 1.24)	1.11 (0.99, 1.23)
0-<25%	(0,0.53)	86	1.0 (reference)	1.0 (reference)	41	1.0 (reference)	1.0 (reference)
25-<50%	(0.53,0.98)	106	1.17 (0.88, 1.56)	1.16 (0.87, 1.55)	44	1.00 (0.65, 1.53)	1.00 (0.65, 1.53)
50-<75%	(0.98,1.84)	103	1.18 (0.88, 1.57)	1.17 (0.88, 1.56)	47	1.14 (0.75, 1.73)	1.13 (0.74, 1.72)
75-<90%	(1.84,3.46)	66	1.28 (0.93, 1.77)	1.27 (0.92, 1.76)	39	1.57 (1.01, 2.44)	1.54 (0.99, 2.40)
>=90%	(3.46,100)	41	1.24 (0.85, 1.79)	1.22 (0.84, 1.78)	21	1.32 (0.78, 2.23)	1.30 (0.77, 2.21)
P trend[3]			0.26	0.29		0.08	0.09
**TTHM**, N=58207, total person-years=1106228							
Log2		404	0.99 (0.95, 1.02)	0.99 (0.95, 1.03)	194	0.98 (0.93, 1.04)	0.98 (0.93, 1.04)
0-<25%	(0,1.09)	105	1.0 (reference)	1.0 (reference)	53	1.0 (reference)	1.0 (reference)

25-<50%	(1.10,6.83)	94	0.82 (0.62, 1.08)	0.82 (0.62, 1.09)	40	0.69 (0.46, 1.05)	0.70 (0.46, 1.06)
50-<75%	(6.83,19.04)	96	0.85 (0.64, 1.12)	0.85 (0.64, 1.13)	48	0.84 (0.57, 1.24)	0.85 (0.57, 1.26)
75-<90%	(19.04,28.18)	67	0.95 (0.70, 1.29)	0.95 (0.70, 1.30)	33	0.93 (0.60, 1.44)	0.94 (0.61, 1.46)
>=90%	(28.19,172.05)	42	0.93 (0.65, 1.33)	0.93 (0.65, 1.34)	20	0.89 (0.53, 1.49)	0.90 (0.53, 1.50)
P trend[3]			0.95	0.92		0.82	0.80

[1]Model 1 was adjusted for age (time-varying) + age^2.
[2]Model 2 = model 1 + BMI category (<25 kg/m^2, 25-<30 kg/m^2, >30 kg/m^2, or missing), menopause status (pre-, peri/post-menopause, or missing), live births (yes/no/missing), oral contraceptive use (never/ever/missing).
[3]P trend was evaluated using the median of each quantile.

Table S5. Hazard ratios (95% CI) for all epithelial ovarian carcinomas and high grade serous ovarian cancers per doubling of community water system (CWS) exposures among California Teachers Study participants, by menopause and body mass index (BMI) category. CWS exposures[1] were linked to the address at enrollment. Analyses were restricted to participants with a residential duration at enrollment ≥ 10 years[2]. Statistical interaction by BMI was observed for arsenic for epithelial cancer risk only ($p<0.05$); no statistical interaction was observed in all other analyses.

		All Epithelial Carcinomas			High-grade Serous Histotype		
	N	N cases	HR (95% CI) Model 1[3]	HR (95% CI) Model 2[4]	N Cases	HR (95% CI) Model 1[3]	HR (95% CI) Model 2[4]
**By menopause status**							
**Nitrate-N (mg/L)**							
Pre	25349	136	1.03 (0.95, 1.13)	1.03 (0.95, 1.12)	62	1.08 (0.95, 1.22)	1.07 (0.95, 1.22)
Peri/Post	28699	231	1.03 (0.96, 1.10)	1.03 (0.96, 1.09)	112	1.10 (1.00, 1.21)	1.10 (1.00, 1.21)
**Uranium (µg/L)**							
Pre	23766	127	1.11 (0.99, 1.25)	1.11 (0.98, 1.25)	60	1.06 (0.89, 1.25)	1.05 (0.89, 1.25)
Peri/Post	27087	220	1.07 (0.98, 1.17)	1.07 (0.98, 1.16)	109	1.07 (0.94, 1.21)	1.06 (0.94, 1.20)
**Arsenic (µg/L)**							
Pre	25349	136	1.08 (0.94, 1.24)	1.08 (0.94, 1.24)	62	1.13 (0.92, 1.38)	1.12 (0.91, 1.37)
Peri/Post	28699	231	1.02 (0.92, 1.14)	1.02 (0.91, 1.14)	112	1.02 (0.87, 1.20)	1.02 (0.87, 1.20)
**TTHM (µg/L)**							
Pre	25349	136	1.00 (0.94, 1.05)	1.00 (0.94, 1.05)	62	0.99 (0.91, 1.07)	0.99 (0.92, 1.07)
Peri/Post	28699	231	1.02 (0.97, 1.06)	1.02 (0.97, 1.06)	112	1.02 (0.96, 1.09)	1.03 (0.96, 1.09)
**By BMI category**							
**Nitrate-N (mg/L)**							
<25 kg/m^2	34878	239	1.02 (0.96, 1.09)	1.02 (0.96, 1.09)	110	1.08 (0.99, 1.19)	1.08 (0.98, 1.19)
25-<30 kg/m^2	14455	104	1.02 (0.93, 1.13)	1.02 (0.93, 1.12)	56	1.07 (0.93, 1.22)	1.06 (0.93, 1.21)
>=30 kg/m^2	8216	59	1.05 (0.92, 1.20)	1.05 (0.92, 1.20)	27	1.10 (0.91, 1.34)	1.10 (0.90, 1.34)
**Uranium (µg/L)**							
<25 kg/m^2	32765	227	1.08 (0.99, 1.17)	1.07 (0.98, 1.17)	108	1.06 (0.93, 1.20)	1.06 (0.93, 1.20)
25-<30 kg/m^2	13608	100	1.06 (0.93, 1.21)	1.06 (0.93, 1.21)	55	0.99 (0.84, 1.18)	0.98 (0.83, 1.17)
>=30 kg/m^2	7741	55	1.11 (0.93, 1.33)	1.11 (0.92, 1.32)	25	1.05 (0.81, 1.36)	1.05 (0.80, 1.36)
**Arsenic (µg/L)**							
<25 kg/m^2	34878	239	0.99 (0.88, 1.10)	0.98 (0.88, 1.10)	110	1.05 (0.89, 1.23)	1.05 (0.89, 1.23)
25-<30 kg/m^2	14455	104	1.28 (1.10, 1.49)	1.28 (1.10, 1.49)	56	1.20 (0.98, 1.49)	1.20 (0.97, 1.48)
>=30 kg/m^2	8216	59	0.98 (0.79, 1.21)	0.97 (0.78, 1.21)	27	0.98 (0.71, 1.34)	0.98 (0.71, 1.34)
**TTHM (µg/L)**							
<25 kg/m^2	34878	239	1.00 (0.96, 1.05)	1.01 (0.96, 1.05)	110	1.01 (0.95, 1.07)	1.01 (0.95, 1.07)

25-<30 kg/m^2	14455	104	1.01 (0.95, 1.08)	1.02 (0.95, 1.08)	56	1.05 (0.96, 1.14)	1.05 (0.96, 1.15)
>=30 kg/m^2	8216	59	1.03 (0.94, 1.12)	1.03 (0.94, 1.12)	27	1.00 (0.89, 1.14)	1.01 (0.89, 1.14)

[1]CWS concentrations represent 15-year average (1990-2005) concentrations of arsenic (µg/L), uranium (µg/L), nitrate-N (mg/L), and TTHM (µg/L).
[2]Residential duration at the enrollment address was determined based on the residential history and self-reported information about duration at the current home at questionnaire 4. Mean follow-up=19.0 years, total person-years: 1,139,582. For uranium, mean follow-up=19.0 years, total person-years: 1,072,419.
[3]Model 1 was adjusted for baseline age (years) + baseline age^2.
[4]Model 2 = model 1 + BMI category (<25 kg/m^2, 25-<30 kg/m^2, >30 kg/m^2, or missing), menopause status (pre-, peri/post-menopause, or missing), live births (yes/no/missing), oral contraceptive use (never/ever/missing). Models stratified by BMI or menopause were not adjusted for BMI or menopause status, respectively.

Table S6. Hazard ratios (95% CI) for all epithelial ovarian carcinomas and high grade serous ovarian cancers per doubling of community water system (CWS) exposures among California Teachers Study participants, by smoking status. CWS exposures[1] were linked to the address at enrollment. Analyses were restricted to participants with a residential duration at enrollment ≥ 10 years[2]. No statistically significant interaction was observed (p>0.05) with smoking status for any contaminants.

		All Epithelial Carcinomas			High-grade Serous Histotype		
**By smoking status**	**N**	**N cases**	**HR (95% CI) Model 1[3]**	**HR (95% CI) Model 2[4]**	**N Cases**	**HR (95% CI) Model 1[3]**	**HR (95% CI) Model 2[4]**
**Nitrate-N (mg/L)**							
Never	39731	267	1.04 (0.98, 1.11)	1.04 (0.98, 1.10)	128	1.11 (1.01, 1.21)	1.10 (1.01, 1.21)
Former	16818	130	1.02 (0.94, 1.11)	1.02 (0.94, 1.11)	65	1.06 (0.94, 1.20)	1.06 (0.94, 1.20)
Current	2976	14	0.98 (0.75, 1.27)	0.99 (0.76, 1.29)	6	1.08 (0.71, 1.63)	1.09 (0.72, 1.65)
**Uranium (µg/L)**							
Never	37351	249	1.08 (0.99, 1.18)	1.08 (0.99, 1.17)	124	1.03 (0.92, 1.16)	1.03 (0.92, 1.16)
Former	15814	127	1.10 (0.98, 1.23)	1.09 (0.97, 1.22)	64	1.08 (0.92, 1.27)	1.07 (0.91, 1.26)
Current	2809	14	0.99 (0.70, 1.40)	1.00 (0.71, 1.41)	6	1.05 (0.61, 1.80)	1.07 (0.62, 1.83)
**Arsenic (µg/L)**							
Never	39731	267	1.07 (0.97, 1.19)	1.07 (0.96, 1.18)	128	1.12 (0.97, 1.29)	1.11 (0.96, 1.28)
Former	16818	130	1.07 (0.92, 1.24)	1.06 (0.91, 1.23)	65	1.05 (0.85, 1.29)	1.04 (0.84, 1.28)
Current	2976	14	0.75 (0.46, 1.22)	0.77 (0.47, 1.26)	6	0.53 (0.24, 1.20)	0.54 (0.24, 1.22)
**TTHM (µg/L)**							
Never	39731	267	1.00 (0.97, 1.04)	1.01 (0.97, 1.05)	128	1.03 (0.97, 1.09)	1.03 (0.97, 1.09)
Former	16818	130	1.02 (0.96, 1.08)	1.02 (0.96, 1.08)	65	0.98 (0.91, 1.06)	0.99 (0.91, 1.07)
Current	2976	14	0.98 (0.83, 1.15)	0.97 (0.82, 1.14)	6	0.97 (0.75, 1.25)	0.96 (0.74, 1.24)

[1]CWS concentrations represent 15-year average (1990-2005) concentrations of arsenic (µg/L), uranium (µg/L), nitrate-N (mg/L), and TTHM (µg/L).
[2]Residential duration at the enrollment address was determined based on the residential history and self-reported information about duration at the current home at questionnaire 4.
[3]Model 1 was adjusted for baseline age (years) + baseline age^2.
[4]Model 2 = model 1 + BMI category (<25 kg/m^2, 25-<30 kg/m^2, >30 kg/m^2, or missing), menopause status (pre-, peri/post-menopause, or missing), live births (yes/no/missing), oral contraceptive use (never/ever/missing).

Table S7. Hazard ratios (95% CI) for all epithelial ovarian carcinomas and high grade serous ovarian cancers per doubling of community water system (CWS) exposures, and per interquartile range increase in the contaminant mixture, among California Teachers Study participants stratified by urbanicity.[1] CWS exposures were linked to the address at enrollment. Analyses were restricted to participants with a residential duration at enrollment ≥ 10 years[2]. No statistical interaction by urbanicity was observed for arsenic, uranium, or TTHM (P trend for interaction ≥0.05). Statistical interaction was observed for nitrate for high-grade serous cancer risk (p<0.05 in models 1 and 2).

			All Epithelial Carcinomas				High-grade Serous Histotype			
	Median (IQR)	N	N Cases	HR (95% CI) Model 1[3]	HR (95% CI) Model 2[4]	HR (95% CI) Model 3[5]	N Cases	HR (95% CI) Model 1[3]	HR (95% CI) Model 2[4]	HR (95% CI) Model 3[5]
**Not metropolitan**										
Nitrate-N (mg/L)	1.09 (0.29, 2.43)	18775	128	1.03 (0.94, 1.14)	1.04 (0.94, 1.14)	1.01 (0.91, 1.11)	56	1.26 (1.08, 1.48)	1.26 (1.07, 1.48)	1.19 (1.01, 1.41)
Uranium (µg/L)	4.39 (1.67, 8.19)	15727	111	1.09 (0.97, 1.23)	1.10 (0.98, 1.23)	1.06 (0.94, 1.19)	54	1.13 (0.95, 1.34)	1.13 (0.96, 1.34)	1.09 (0.92, 1.28)
Arsenic (µg/L)	1.64 (0.69, 2.29)	18775	128	1.10 (0.96, 1.25)	1.10 (0.97, 1.25)	1.09 (0.95, 1.25)	56	1.23 (1.01, 1.49)	1.23 (1.01, 1.49)	1.23 (1.00, 1.51)
TTHM (µg/L)	2.51 (0.19, 14.09)	18775	128	1.02 (0.97, 1.08)	1.02 (0.97, 1.08)	1.03 (0.97, 1.10)	56	1.02 (0.93, 1.11)	1.02 (0.94, 1.11)	1.04 (0.94, 1.15)
**Mixture[6]**		15727	111	1.64 (0.80, 3.34)	1.64 (0.81, 3.33)	1.76 (0.81, 3.84)	58	4.65 (1.59, 13.58)	4.69 (1.60, 13.70)	5.80 (1.70, 19.78)
**Metropolitan**										
Nitrate-N (mg/L)	0.43 (0.18, 2.11)	40777	282	1.03 (0.97, 1.09)	1.02 (0.97, 1.08)	1.02 (0.95, 1.1)	141	1.06 (0.97, 1.15)	1.05 (0.97, 1.14)	1.02 (0.93, 1.13)
Uranium (µg/L)	3.69 (1.01, 5.99)	40291	278	1.06 (0.98, 1.15)	1.06 (0.98, 1.15)	1.06 (0.97, 1.16)	138	1.01 (0.9, 1.13)	1.01 (0.90, 1.13)	0.97 (0.86, 1.10)
Arsenic (µg/L)	0.97 (0.59, 1.62)	40777	282	1.02 (0.91, 1.14)	1.02 (0.91, 1.13)	1.03 (0.9, 1.17)	141	1.02 (0.88, 1.2)	1.02 (0.87, 1.19)	0.99 (0.82, 1.19)
TTHM (µg/L)	5.44 (0.79, 21.49)	40777	282	1.00 (0.96, 1.04)	1.00 (0.96, 1.04)	1.00 (0.96, 1.04)	141	1.00 (0.95, 1.06)	1.00 (0.95, 1.06)	1.03 (0.96, 1.09)
**Mixture[6]**		40291	278	1.22 (0.86, 1.74)	1.22 (0.85, 1.74)	1.24 (0.80, 1.91)	138	1.34 (0.81, 2.21)	1.33 (0.80, 2.20)	1.30 (0.70, 2.40)

[1]Census-defined Urbanized Areas were assigned to census Blocks; metropolitan urban areas were defined as having a population ≥1,000,000 and non-metropolitan city urban areas were defined as population <1,000,000. Population information from census Places were used to further describe census Blocks as a city (population ≥50,000) or town (population <50,000). Census Blocks that were not assigned to the before-mentioned categories were classified as rural. Census Blocks were aggregated to census Block Groups per the following process: Block Groups consisting of Blocks with the same urban code were categorized as that code; Block Groups consisting of Blocks with different urban codes were assigned an urban code based on the population distribution, such that if any urban code accounted for >50% of the population then that urban code was assigned to the Block Group. For Block Groups that remained unassigned, the highest-ranking urban code (that accounted for ≥25% of the population) was assigned. Additional adjustments were made to incorporate population density information: Block Groups coded as a metropolitan urban area with a population density in the top quartile retained their metropolitan urban code, and the remaining metropolitan Block Groups (in the lower three quartiles of population density) were re-categorized as metropolitan suburban. Block Groups that were categorized as towns were re-categorized to rural if their population density was in the lowest quartile. We dichotomized urbanization categories as non-metropolitan (rural, town, city), and metropolitan (metropolitan suburban and metropolitan urban).[7,8]

[2]CWS concentrations represent 15-year average (1990-2005) concentrations of arsenic (µg/L), uranium (µg/L), nitrate-N (mg/L), and TTHM (µg/L). Residential duration at the enrollment address was determined based on the residential history and self-reported information about duration at the current home at questionnaire 4. For non-metropolitan participants, mean follow-up=18.9 years, total person-years: 355,743. For metropolitan participants, mean follow-up=19.1 years, total person-years: 777,113.8.

[3]Model 1 was adjusted for baseline age (years) + baseline age^2.

[4]Model 2 = model 1 + BMI category (<25 kg/m^2, 25-<30 kg/m^2, >30 kg/m^2, or missing), menopause status (pre-, peri/post-menopause, or missing), live births (yes/no/missing), oral contraceptive use (never/ever/missing).
[5]Model 3 = model 2 + size of population served (very small (≤500 people), small (>500–3300 people), medium (>3300–10,000 people), large (>10,000–<1,000,000 people), and very large (≥1,000,000 people) and water source (groundwater, surface water, groundwater under the influence of surface water).
[6]Concentrations of the four contaminants were log2 transformed and subsequently divided by the IQR. The effect of the drinking water contaminant mixture was evaluated using quantile q-computation, and hazards ratios are interpreted per IQR increase.

Table S8. Hazard ratios (95% CI) for all epithelial ovarian carcinomas and high grade serous ovarian cancers per doubling of community water system (CWS) exposures, among California Teachers Study participants stratified by the size of the CWS population served.[1] CWS exposures were linked to the address at enrollment. Analyses were restricted to participants with a residential duration at enrollment ≥ 10 years.[2] No statistical interaction by urbanicity was observed for arsenic, uranium, or TTHM (P for interaction ≥0.05). Statistical interaction was observed for nitrate for epithelial cancer risk only (p<0.05); no statistical interaction was observed in all other analyses.

			All Epithelial Carcinomas				High-grade Serous Histotype			
	**Median (IQR)**	**N**	**N Cases**	**HR (95% CI) Model 1[3]**	**HR (95% CI) Model 2[4]**	**HR (95% CI) Model 3[5]**	**N Cases**	**HR (95% CI) Model 1[3]**	**HR (95% CI) Model 2[4]**	**HR (95% CI) Model 3[5]**
**Very small**										
Nitrate-N (mg/L)	0.8 (0.09,1.98)	342	4	--	--		0	--	--	
Uranium (µg/L)	3.84 (3.84,5.25)	129	3				0			
Arsenic (µg/L)	1.16 (0.44,2.27)	342	4	--	--		0	--	--	
TTHM (µg/L)	0.09 (0.06,0.56)	342	4	--	--		0	--	--	
**Small**										
Nitrate-N (mg/L)	0.52 (0.16,1.36)	705	1	--	--		0	--	--	
Uranium (µg/L)	2.61 (0.77,4.47)	425	1				0			
Arsenic (µg/L)	0.98 (0.46,1.82)	705	1	--	--		0	--	--	
TTHM (µg/L)	1.38 (0.12,8.39)	705	1	--	--		0	--	--	
**Medium**										
Nitrate-N (mg/L)	1.22 (0.26,2.22)	2251	17	1.50 (1.08, 2.09)	1.54 (1.1, 2.16)	1.51 (1.07, 2.14)	10	1.80 (1.08, 2.99)	1.82 (1.09, 3.04)	1.73 (1.02, 2.93)
Uranium (µg/L)	3.42 (0.93,7.16)	1709	13	1.53 (1.09, 2.15)	1.58 (1.12, 2.25)	1.60 (1.11, 2.31)	8	1.42 (0.93, 2.16)	1.41 (0.92, 2.15)	1.46 (0.93, 2.29)
Arsenic (µg/L)	1.04 (0.51,2.18)	2251	17	1.21 (0.88, 1.67)	1.24 (0.89, 1.73)	1.30 (0.87, 1.95)	10	1.37 (0.91, 2.07)	1.41 (0.91, 2.18)	1.40 (0.81, 2.40)
TTHM (µg/L)	0.94 (0.15,8.07)	2251	17	0.96 (0.81, 1.12)	0.97 (0.83, 1.15)	0.99 (0.81, 1.21)	10	0.89 (0.71, 1.11)	0.90 (0.72, 1.12)	0.96 (0.73, 1.26)
**Large**										
Nitrate-N (mg/L)	0.85 (0.24,2.69)	22879	134	0.97 (0.88, 1.06)	0.96 (0.88, 1.05)	0.95 (0.86, 1.05)	63	1.11 (0.97, 1.26)	1.10 (0.96, 1.26)	1.07 (0.93, 1.24)
Uranium (µg/L)	3.64 (1.43,5.39)	20484	121	1.05 (0.92, 1.21)	1.05 (0.92, 1.20)	1.07 (0.93, 1.23)	63	0.95 (0.79, 1.14)	0.95 (0.79, 1.14)	0.94 (0.77, 1.13)
Arsenic (µg/L)	1.05 (0.60,2.07)	22879	134	1.00 (0.87, 1.15)	1.00 (0.87, 1.15)	1.01 (0.87, 1.16)	63	1.00 (0.81, 1.22)	0.99 (0.81, 1.22)	1.02 (0.83, 1.26)
TTHM (µg/L)	2.73 (0.15,13.06)	22879	134	1.03 (0.98, 1.09)	1.03 (0.98, 1.09)	1.04 (0.98, 1.1)	63	1.08 (0.99, 1.17)	1.08 (1.00, 1.17)	1.12 (1.02, 1.23)
**Very large**										
Nitrate-N (mg/L)	0.43 (0.15,2.03)	33704	257	1.06 (1.00, 1.13)	1.06 (1.00, 1.12)	1.05 (0.98, 1.14)	126	1.08 (0.99, 1.17)	1.07 (0.98, 1.17)	1.05 (0.94, 1.17)
Uranium (µg/L)	4.61 (1.35,7.93)	33567	254	1.04 (0.97, 1.13)	1.04 (0.96, 1.12)	1.04 (0.95, 1.14)	123	1.05 (0.94, 1.18)	1.05 (0.94, 1.17)	1.01 (0.89, 1.16)
Arsenic (µg/L)	0.97 (0.59,1.76)	33704	257	1.09 (0.97, 1.22)	1.08 (0.97, 1.21)	1.12 (0.97, 1.28)	126	1.11 (0.94, 1.30)	1.10 (0.94, 1.30)	1.11 (0.91, 1.36)
TTHM (µg/L)	8.07 (1.48,21.49)	33704	257	0.99 (0.95, 1.03)	0.99 (0.95, 1.03)	0.99 (0.95, 1.04)	126	0.97 (0.91, 1.03)	0.97 (0.91, 1.03)	0.98 (0.92, 1.05)

[1]CWS population served was classified as follows: population served (very small (≤500 people), small (>500–3300 people), medium (>3300–10,000 people), large (>10,000–<1,000,000 people), and very large (≥1,000,000 people). Due to a limited number of cases among participants served by very small and small CWS (<5) cases), we did not conduct analyses among those groups.
[2]CWS concentrations represent 15-year average (1990-2005) concentrations of arsenic (μg/L), uranium (μg/L), nitrate-N (mg/L), and TTHM (μg/L). Residential duration at the enrollment address was determined based on the residential history and self-reported information about duration at the current home at questionnaire 4.
[3]Model 1 was adjusted for baseline age (years) + baseline age^2.
[4]Model 2 = model 1 + BMI category (<25 kg/m^2, 25-<30 kg/m^2, >30 kg/m^2, or missing), menopause status (pre-, peri/post-menopause, or missing), live births (yes/no/missing), oral contraceptive use (never/ever/missing).
[5]Model 3 = model 2 + water source (groundwater, surface water, groundwater under the influence of surface water) + urbanicity (metropolitan, non-metropolitan).

Table S9. Hazard ratios (95% CI) for all epithelial ovarian carcinomas and high grade serous ovarian cancers per doubling of community water system (CWS) exposures, and per interquartile range increase in the contaminant mixture, among California Teachers Study participants stratified by CWS water source.[1] CWS exposures were linked to the address at enrollment. Analyses were restricted to participants with a residential duration at enrollment ≥ 10 years[2]. No statistical interaction by water source type was observed in single contaminant analyses (P trend for interaction ≥0.05).

			All Epithelial Carcinomas				High-grade Serous Histotype			
	Median (IQR)	N	N Cases	HR (95% CI) Model 1[3]	HR (95% CI) Model 2[4]	HR (95% CI) Model 3[5]	N Cases	HR (95% CI) Model 1[3]	HR (95% CI) Model 2[4]	HR (95% CI) Model 3[5]
**Ground water**										
Nitrate-N (mg/L)	2.05 (0.78,3.32)	9150	61	1.08 (0.92, 1.28)	1.08 (0.91, 1.27)	1.06 (0.89, 1.27)	32	1.07 (0.85, 1.34)	1.06 (0.84, 1.33)	1.02 (0.80, 1.31)
Uranium (µg/L)	4.96 (1.67,8.22)	7278	50	0.98 (0.84, 1.15)	0.98 (0.83, 1.15)	0.99 (0.84, 1.17)	28	1.00 (0.81, 1.24)	1.01 (0.81, 1.25)	1.02 (0.82, 1.28)
Arsenic (µg/L)	1.2 (0.59,3.34)	9150	61	1.06 (0.90, 1.25)	1.05 (0.89, 1.24)	1.10 (0.92, 1.31)	32	1.13 (0.91, 1.41)	1.13 (0.91, 1.41)	1.23 (0.95, 1.59)
TTHM (µg/L)	0.27 (0.13,0.64)	9150	61	1.08 (0.97, 1.2)	1.07 (0.97, 1.20)	1.07 (0.95, 1.20)	32	1.13 (0.98, 1.30)	1.13 (0.98, 1.30)	1.15 (0.98, 1.35)
**Mixture**[6]				2.36 (0.97, 5.73)	2.29 (0.94, 5.58)	2.37 (0.95, 5.95)		4.96 (1.58, 15.56)	4.90 (1.51, 15.91)	5.18 (1.50, 17.93)
**Surface water**										
Nitrate-N (mg/L)	0.43 (0.16,1.89)	45748	313	1.02 (0.96, 1.08)	1.02 (0.96, 1.08)	1.03 (0.97, 1.09)	145	1.09 (1.01, 1.19)	1.09 (1.00, 1.19)	1.11 (1.02, 1.21)
Uranium (µg/L)	3.42 (1.01,5.11)	44144	303	1.12 (1.03, 1.21)	1.12 (1.03, 1.21)	1.11 (1.02, 1.21)	144	1.06 (0.94, 1.19)	1.05 (0.94, 1.18)	1.05 (0.94, 1.18)
Arsenic (µg/L)	0.97 (0.59,1.75)	45748	313	1.04 (0.94, 1.16)	1.04 (0.94, 1.16)	1.06 (0.95, 1.18)	145	1.03 (0.89, 1.20)	1.03 (0.88, 1.19)	1.06 (0.90, 1.25)
TTHM (µg/L)	7.04 (0.9,20.84)	45748	313	1.00 (0.97, 1.04)	1.00 (0.97, 1.04)	1.00 (0.96, 1.04)	145	1.01 (0.96, 1.07)	1.01 (0.96, 1.07)	1.01 (0.95, 1.06)
**Mixture**[6]				1.32 (0.92, 1.90)	1.33 (0.93, 1.90)	1.33 (0.91, 1.95)		1.66 (0.98, 2.80)	1.64 (0.97, 2.76)	1.65 (0.95, 2.86)
**Groundwater under influence of surface water**										
Nitrate-N (mg/L)	5.02 (1.52,5.02)	1112	9	--	--		8	--	--	--
Uranium (µg/L)	16.22 (11.26,16.22)	1021	9	--	--		8	--	--	--
Arsenic (µg/L)	1.66 (1.39,1.66)	1112	9	--	--		8	--	--	--
TTHM (µg/L)	1.88 (1.83,1.88)	1112	9	--	--		8	--	--	--

[1]CWS water source was classified as follows: groundwater, groundwater under the influence of surface water, and surface water. Due to a limited number of cases among participants served by groundwater under the influence of surface water (<10) cases), we did not conduct analyses among those groups.
[2]CWS concentrations represent 15-year average (1990-2005) concentrations of arsenic (µg/L), uranium (µg/L), nitrate-N (mg/L), and TTHM (µg/L). Residential duration at the enrollment address was determined based on the residential history and self-reported information about duration at the current home at questionnaire 4.
[3]Model 1 was adjusted for baseline age (years) + baseline age^2.
[4]Model 2 = model 1 + BMI category (<25 kg/m^2, 25-<30 kg/m^2, >30 kg/m^2, or missing), menopause status (pre-, peri/post-menopause, or missing), live births (yes/no/missing), oral contraceptive use (never/ever/missing).
[5]Model 3 = Model 2 + size of the population served by the CWS [very small (≤500 people), small (>500–3300 people), medium (>3300–10,000 people), large (>10,000–<1,000,000 people), and very large (≥1,000,000 people)] + urbanicity (metropolitan, non metropolitan).

[6]Concentrations were log2 transformed and subsequently divided by the IQR. The effect of the drinking water contaminant mixture was evaluated using quantile q-computation, and hazards ratios are interpreted per IQR increase.

Table S10-A. Hazard ratios (95% CI) for all epithelial ovarian carcinomas per doubling and quartile of community water system (CWS) exposures among California Teachers Study participants, stratified by tertile of by vitamin C intake (mg/day). CWS exposures[1] were linked to the address at enrollment. Analyses were restricted to participants with a residential duration at enrollment ≥ 10 years[2], and total daily vitamin C and caloric intake (N=54,630).[3]

All Epithelial Carcinomas HR (95% CI)

	Vitamin C Tertile 1 (1.3 - 132.7 mg/day)				Vitamin C Tertile 2 (132.7 - 306.9 mg/day)				Vitamin C Tertile 3 (306.9 - 3446.2 mg/day)				
	N	Cases	Model 1[4]	Model 2[5]	N	Cases	Model 1[4]	Model 2[5]	N	Cases	Model 1[4]	Model 2[5]	P int[6]
**Nitrate-N**	17804	98	1.1 (1, 1.22)	1.11 (1, 1.22)	18735	125	1.05 (0.96, 1.15)	1.05 (0.96, 1.15)	18091	166	1.01 (0.94, 1.09)	1.01 (0.93, 1.09)	0.37
0-<25%	4143	15	1.0 (ref)	1.0 (ref)	4697	28	1.0 (ref)	1.0 (ref)	4584	40	1.0 (ref)	1.0 (ref)	
25-<50%	4461	24	1.49 (0.78, 2.84)	1.51 (0.79, 2.87)	4592	29	1.06 (0.63, 1.78)	1.06 (0.63, 1.78)	4439	42	1.08 (0.7, 1.66)	1.07 (0.69, 1.65)	
50-<75%	4513	32	2.07 (1.12, 3.83)	2.09 (1.13, 3.86)	4824	33	1.19 (0.72, 1.96)	1.17 (0.71, 1.94)	4710	46	1.14 (0.75, 1.75)	1.13 (0.74, 1.73)	
>=75%	4687	27	1.65 (0.88, 3.11)	1.67 (0.89, 3.14)	4622	35	1.32 (0.8, 2.17)	1.31 (0.8, 2.16)	4358	38	1.03 (0.66, 1.61)	1.01 (0.65, 1.58)	
P trend[7]			0.33	0.33			0.26	0.26			0.98	0.92	
**Uranium**	16722	90	1.22 (1.06, 1.41)	1.22 (1.05, 1.41)	17617	120	1.1 (0.98, 1.24)	1.1 (0.98, 1.24)	16994	159	1.02 (0.92, 1.14)	1.02 (0.92, 1.13)	0.14
0-<25%	3926	12	1.0 (ref)	1.0 (ref)	4447	25	1.0 (ref)	1.0 (ref)	4207	36	1.0 (ref)	1.0 (ref)	
25-<50%	4365	21	1.66 (0.81, 3.37)	1.65 (0.81, 3.36)	4411	31	1.3 (0.77, 2.2)	1.29 (0.76, 2.19)	4376	42	1.17 (0.75, 1.82)	1.16 (0.74, 1.81)	
50-<75%	4186	29	2.4 (1.22, 4.71)	2.38 (1.21, 4.67)	4540	32	1.33 (0.79, 2.24)	1.32 (0.78, 2.23)	4155	42	1.22 (0.78, 1.91)	1.2 (0.77, 1.88)	
>=75%	4245	28	2.25 (1.14, 4.43)	2.23 (1.13, 4.38)	4219	32	1.42 (0.84, 2.39)	1.41 (0.83, 2.37)	4256	39	1.1 (0.7, 1.73)	1.08 (0.69, 1.71)	
P trend[7]			0.02	0.03			0.24	0.25			0.76	0.82	
**Arsenic**	17804	98	1.15 (0.98, 1.35)	1.14 (0.97, 1.34)	18735	125	1.17 (1.01, 1.35)	1.17 (1.01, 1.35)	18091	166	0.97 (0.85, 1.11)	0.97 (0.85, 1.11)	0.13
0-<25%	4148	18	1.0 (ref)	1.0 (ref)	4358	22	1.0 (ref)	1.0 (ref)	4275	41	1.0 (ref)	1.0 (ref)	
25-<50%	4580	26	1.34 (0.74, 2.45)	1.35 (0.74, 2.45)	4806	31	1.34 (0.77, 2.31)	1.34 (0.77, 2.31)	4696	38	0.85 (0.55, 1.32)	0.84 (0.54, 1.31)	
50-<75%	4283	26	1.42 (0.78, 2.6)	1.44 (0.79, 2.62)	4639	30	1.33 (0.77, 2.31)	1.32 (0.76, 2.3)	4576	53	1.21 (0.81, 1.82)	1.2 (0.8, 1.81)	
>=75%	4793	28	1.41 (0.78, 2.55)	1.39 (0.77, 2.52)	4932	42	1.8 (1.08, 3.02)	1.78 (1.06, 2.99)	4544	34	0.81 (0.51, 1.27)	0.79 (0.5, 1.25)	
P trend[7]			0.38	0.41			0.03	0.03			0.52	0.48	

[1]CWS concentrations represent 15-year average (1990-2005) concentrations.
[2]Residential duration at the enrollment address was determined based on the residential history and self-reported information about duration at the current home at questionnaire 4. Mean follow-up=19.1 years, total person-years: 1,045,277. For uranium, mean follow-up=19.1 years, total person-years: 982,731.7
[3]Vitamin C (mg/day) and total caloric intake (kilocalories/day) were computed based on the food frequency questionnaire at survey 1.
[4]Model 1 was adjusted for baseline age (years) + baseline age^2 + natural log transformed total daily caloric intake (kilocalories/day).
[5]Model 2 = model 1 + BMI category (<25 kg/m^2, 25-<30 kg/m^2, >30 kg/m^2, or missing), menopause status (pre-, peri/post-menopause, or missing), live births (yes/no/missing), oral contraceptive use (never/ever/missing).
[6]P for interaction (P int) was evaluated using the likelihood ratio test Chi squared test statistic.
[7]P trend was evaluated using the median of each quantile.

Table S10-B. Hazard ratios (95% CI) for high grade serous ovarian cancers per doubling and quartile of community water system (CWS) exposures among California Teachers Study participants, stratified by tertile of by vitamin C intake (mg/day). CWS exposures[1] were linked to the address at enrollment. Analyses were restricted to participants with a residential duration at enrollment ≥ 10 years[2], and total daily vitamin C and caloric intake (N=54,630).[3]

High-grade Serous Histotype HR (95% CI)

	Vitamin C Tertile 1 (1.3 - 132.7 mg/day)				Vitamin C Tertile 2 (132.7 - 306.9 mg/day)				Vitamin C Tertile 3 (306.9 - 3446.2 mg/day)				
	N	Cases	Model 1[4]	Model 2[5]	N	Cases	Model 1[4]	Model 2[5]	N	Cases	Model 1[4]	Model 2[5]	P int[6]
**Nitrate-N**	17804	49	1.15 (0.99, 1.32)	1.14 (0.98, 1.31)	18735	66	1.13 (1, 1.28)	1.13 (1, 1.29)	18091	77	1.08 (0.97, 1.21)	1.08 (0.97, 1.21)	0.78
0-<25%	4143	6	1.0 (ref)	1.0 (ref)	4697	13	1.0 (ref)	1.0 (ref)	4584	17	1.0 (ref)	1.0 (ref)	
25-<50%	4461	11	1.71 (0.63, 4.62)	1.64 (0.61, 4.44)	4592	13	1.02 (0.47, 2.2)	1.03 (0.48, 2.22)	4439	15	0.91 (0.45, 1.81)	0.92 (0.46, 1.85)	
50-<75%	4513	17	2.79 (1.1, 7.07)	2.66 (1.05, 6.76)	4824	16	1.24 (0.59, 2.57)	1.22 (0.59, 2.54)	4710	24	1.4 (0.75, 2.6)	1.41 (0.75, 2.62)	
>=75%	4687	15	2.33 (0.9, 6.01)	2.2 (0.85, 5.68)	4622	24	1.93 (0.98, 3.79)	1.95 (0.99, 3.83)	4358	21	1.34 (0.71, 2.54)	1.37 (0.72, 2.61)	
P trend[7]			0.18	0.22			0.02	0.02			0.27	0.24	
**Uranium**	16722	45	1.09 (0.9, 1.33)	1.08 (0.89, 1.31)	17617	66	1.16 (0.98, 1.37)	1.16 (0.98, 1.37)	16994	76	0.99 (0.86, 1.15)	0.99 (0.86, 1.15)	0.38
0-<25%	3926	10	1.0 (ref)	1.0 (ref)	4447	12	1.0 (ref)	1.0 (ref)	4207	17	1.0 (ref)	1.0 (ref)	
25-<50%	4365	11	1.06 (0.45, 2.5)	1.01 (0.43, 2.39)	4411	18	1.56 (0.75, 3.25)	1.55 (0.75, 3.23)	4376	21	1.24 (0.65, 2.34)	1.23 (0.65, 2.33)	
50-<75%	4186	10	1.01 (0.42, 2.43)	0.98 (0.41, 2.35)	4540	17	1.46 (0.69, 3.05)	1.45 (0.69, 3.05)	4155	21	1.29 (0.68, 2.45)	1.29 (0.68, 2.44)	
>=75%	4245	14	1.37 (0.61, 3.09)	1.29 (0.57, 2.92)	4219	19	1.76 (0.85, 3.62)	1.75 (0.85, 3.62)	4256	17	1.01 (0.51, 1.98)	1 (0.51, 1.97)	
P trend[7]			0.42	0.48			0.18	0.18			0.91	0.90	
**Arsenic**	17804	49	1.27 (1.02, 1.58)	1.25 (1, 1.56)	18735	66	1.12 (0.92, 1.37)	1.12 (0.92, 1.37)	18091	77	1 (0.83, 1.22)	1 (0.83, 1.22)	0.31
0-<25%	4148	10	1.0 (ref)	1.0 (ref)	4358	13	1.0 (ref)	1.0 (ref)	4275	19	1.0 (ref)	1.0 (ref)	
25-<50%	4580	10	0.94 (0.39, 2.27)	0.93 (0.39, 2.24)	4806	13	0.94 (0.44, 2.03)	0.95 (0.44, 2.06)	4696	16	0.77 (0.4, 1.5)	0.79 (0.4, 1.53)	
50-<75%	4283	11	1.09 (0.46, 2.57)	1.08 (0.46, 2.55)	4639	17	1.28 (0.62, 2.63)	1.27 (0.62, 2.62)	4576	25	1.23 (0.68, 2.24)	1.23 (0.68, 2.23)	
>=75%	4793	18	1.67 (0.77, 3.61)	1.62 (0.75, 3.51)	4932	23	1.65 (0.84, 3.26)	1.65 (0.83, 3.26)	4544	17	0.87 (0.45, 1.68)	0.88 (0.46, 1.7)	
P trend[7]			0.10	0.12			0.07	0.07			0.93	0.93	

[1]CWS concentrations represent 15-year average (1990-2005) concentrations of arsenic (μg/L), uranium (μg/L), nitrate-N (mg/L), and TTHM (μg/L).
[2]Residential duration at the enrollment address was determined based on the residential history and self-reported information about duration at the current home at questionnaire 4. Mean follow-up=19.1 years, total person-years: 1,045,277. For uranium, mean follow-up=19.1 years, total person-years: 982,731.7.
[3]Vitamin C (mg/day) and total caloric intake (kilocalories/day) were computed based on the food frequency questionnaire at survey 1.
[4]Model 1 was adjusted for baseline age (years) + baseline age^2 + natural log transformed total daily caloric intake (kilocalories/day).
[5]Model 2 = model 1 + BMI category (<25 kg/m^2, 25-<30 kg/m^2, >30 kg/m^2, or missing), menopause status (pre-, peri/post-menopause, or missing), live births (yes/no/missing), oral contraceptive use (never/ever/missing).
[6]P trend for interaction was evaluated using the likelihood ratio test Chi squared test statistic.
[7]P trend was evaluated using the median of each quantile.

Table S11. Hazard ratios (95% CI) for all epithelial ovarian carcinomas and high grade serous ovarian cancers per doubling and quartile of community water system (CWS) exposures among California Teachers Study participants, stratified by tertile of by total red meat intake (g/day). CWS exposures[1] were linked to the address at enrollment. Analyses were restricted to participants with a residential duration at enrollment ≥ 10 years[2], and total red meat and caloric intake (N=54,630).[3]

**All Epithelial Carcinomas HR (95% CI)**													
			**Red Meat T1, (0.1 - 18.9 g/day)**				**Red Meat T2, (19.0 - 41.1 g/day)**				**Red Meat T3, (41.2 - 326.5 g/day)**		
**Nitrate-N**	**N**	**Cases**	**Model 1[4]**	**Model 2[5]**	**N**	**Cases**	**Model 1[4]**	**Model 2[5]**	**N**	**Cases**	**Model 1[4]**	**Model 2[5]**	**P int[6]**
Log2	17974	125	0.98 (0.90, 1.07)	0.98 (0.90, 1.07)	18616	145	1.12 (1.03, 1.22)	1.12 (1.03, 1.22)	18040	119	1.02 (0.94, 1.12)	1.02 (0.93, 1.12)	0.07
0-<25%	5063	41	1.0 (ref)	1.0 (ref)	4466	22	1.0 (ref)	1.0 (ref)	3895	20	1.0 (ref)	1.0 (ref)	
25-<50%	4619	31	0.83 (0.52, 1.33)	0.84 (0.53, 1.34)	4561	34	1.48 (0.86, 2.53)	1.47 (0.86, 2.51)	4312	30	1.37 (0.78, 2.42)	1.38 (0.78, 2.43)	
50-<75%	4452	24	0.68 (0.41, 1.12)	0.68 (0.41, 1.13)	4798	47	2.03 (1.22, 3.36)	2.02 (1.21, 3.35)	4797	40	1.73 (1.01, 2.97)	1.73 (1.01, 2.97)	
>=75%	3840	29	0.95 (0.59, 1.53)	0.95 (0.59, 1.54)	4791	42	1.83 (1.09, 3.07)	1.82 (1.09, 3.06)	5036	29	1.18 (0.67, 2.10)	1.18 (0.67, 2.09)	
P trend[7]			0.92	0.92			0.08	0.08			0.87	0.84	
**High-grade Serous Histotype HR (95% CI)**													
			**Red Meat T1, (0.1 - 18.9 g/day)**				**Total Red Meat T2, (19.0 - 41.1 g/day)**				**Red Meat T3, (41.2 - 326.5 g/day)**		
**Nitrate-N**	**N**	**Cases**	**Model 1[4]**	**Model 2[5]**	**N**	**Cases**	**Model 1[4]**	**Model 2[5]**	**N**	**Cases**	**Model 1[4]**	**Model 2[5]**	**P int[6]**
Log2	17974	61	1.08 (0.96, 1.23)	1.09 (0.96, 1.23)	18616	77	1.16 (1.03, 1.30)	1.16 (1.03, 1.30)	18040	54	1.09 (0.95, 1.25)	1.09 (0.95, 1.25)	0.68
0-<25%	5063	17	1.0 (ref)	1.0 (ref)	4466	13	1.0 (ref)	1.0 (ref)	3895	6	1.0 (ref)	1.0 (ref)	
25-<50%	4619	13	0.84 (0.41, 1.74)	0.86 (0.42, 1.77)	4561	14	1.04 (0.49, 2.20)	1.02 (0.48, 2.16)	4312	12	1.83 (0.69, 4.88)	1.84 (0.69, 4.91)	
50-<75%	4452	13	0.89 (0.43, 1.83)	0.90 (0.44, 1.86)	4798	24	1.76 (0.90, 3.46)	1.72 (0.88, 3.39)	4797	20	2.90 (1.17, 7.24)	2.90 (1.16, 7.24)	
>=75%	3840	18	1.43 (0.74, 2.77)	1.46 (0.75, 2.84)	4791	26	1.94 (1.00, 3.78)	1.90 (0.97, 3.71)	5036	16	2.19 (0.86, 5.61)	2.19 (0.85, 5.60)	
P trend[7]			0.15	0.14			0.03	0.03			0.31	0.32	

[1]CWS concentrations represent 15-year average (1990-2005) concentrations of nitrate-N (mg/L.

[2]Residential duration at the enrollment address was determined based on the residential history and self-reported information about duration at the current home at questionnaire 4. Mean follow-up=19.1 years, total person-years: 1,045,277.

[3]Red meat (g/day) and total caloric intake (kilocalories/day) were computed based on the food frequency questionnaire at survey 1. Zero values were imputed as the lowest non-zero estimate / 10; zero values were imputed as 0.1 g/day.

[4]Model 1 was adjusted for baseline age (years) + baseline age^2 + natural log transformed total daily caloric intake (kilocalories/day).

[5]Model 2 = model 1 + BMI category (<25 kg/m^2, 25-<30 kg/m^2, >30 kg/m^2, or missing), menopause status (pre-, peri/post-menopause, or missing), live births (yes/no/missing), oral contraceptive use (never/ever/missing).

[6]P trend for interaction was evaluated using the likelihood ratio test Chi squared test statistic.

[7]P trend was evaluated using the median of each quantile.

Table S12. Hazard ratios (95% CI) for all epithelial ovarian carcinomas and high grade serous ovarian cancers per doubling and quartile (Q) of dietary nitrate and nitrite intake (mg/day) among California Teachers Study participants. CWS exposures[1] were linked to the address at enrollment. Analyses were restricted to participants with a residential duration at enrollment ≥ 10 years[2], and dietary nitrate, nitrite, and caloric intake (N=54,630).[3]

			All Epithelial Carcinomas			High-grade Serous Histotype		
	Concentration Range (mg/day)	N	N Cases	HR (95% CI) Model 1[4]	HR (95% CI) Model 2[5]	N Cases	HR (95% CI) Model 1[4]	HR (95% CI) Model 2[5]
**Total nitrate**	Log2	54630	389	1.04 (0.91, 1.19)	1.04 (0.91, 1.19)	192	0.99 (0.82, 1.20)	0.99 (0.82, 1.20)
0-<25%	4.26, 44.95	10078	61	1.0 (reference)	1.0 (reference)	31	1.0 (reference)	1.0 (reference)
25-<50%	44.96, 72.54	14780	103	1.07 (0.78, 1.48)	1.08 (0.78, 1.49)	49	0.98 (0.62, 1.55)	0.98 (0.62, 1.54)
50-<75%	72.55, 106.57	14871	110	1.07 (0.77, 1.48)	1.08 (0.78, 1.49)	57	1.06 (0.67, 1.67)	1.06 (0.67, 1.67)
>=75%	106.58, 500.41	14901	115	1.06 (0.75, 1.48)	1.07 (0.76, 1.50)	55	0.95 (0.59, 1.53)	0.96 (0.59, 1.55)
P trend[7]				0.86	0.81		0.83	0.87
**Total nitrite**	Log2	54630	389	1.16 (0.88, 1.53)	1.16 (0.88, 1.52)	192	1.27 (0.86, 1.88)	1.29 (0.87, 1.91)
0-<25%	0.13, 0.79	9804	66	1.0 (reference)	1.0 (reference)	30	1.0 (reference)	1.0 (reference)
25-<50%	0.79, 1.10	14936	98	0.98 (0.70, 1.37)	0.98 (0.70, 1.36)	47	1.05 (0.65, 1.72)	1.06 (0.65, 1.73)
50-<75%	1.10, 1.46	14962	117	1.18 (0.82, 1.7)	1.18 (0.82, 1.70)	57	1.31 (0.77, 2.24)	1.33 (0.78, 2.26)
>=75%	1.46, 5.68	14928	108	1.12 (0.72, 1.74)	1.11 (0.72, 1.74)	58	1.41 (0.75, 2.65)	1.42 (0.76, 2.69)
P trend[7]				0.49	0.51		0.22	0.21
**Plant nitrite**	Log2	54630	389	1.02 (0.83, 1.26)	1.02 (0.83, 1.27)	192	1.13 (0.84, 1.53)	1.14 (0.84, 1.55)
0-<25%	0.05, 0.55	9841	73	1.0 (reference)	1.0 (reference)	33	1.0 (reference)	1.0 (reference)
25-<50%	0.55, 0.77	14921	98	0.85 (0.62, 1.17)	0.85 (0.62, 1.17)	45	0.9 (0.56, 1.43)	0.9 (0.56, 1.44)
50-<75%	0.77, 1.05	14946	109	0.93 (0.66, 1.30)	0.93 (0.66, 1.31)	52	1.04 (0.63, 1.70)	1.04 (0.63, 1.71)
>=75%	1.05, 4.56	14922	109	0.90 (0.61, 1.33)	0.90 (0.61, 1.33)	62	1.24 (0.72, 2.14)	1.25 (0.73, 2.17)
P trend[7]				0.82	0.82		0.25	0.24
**Animal nitrite**	Log2	54630	389	1.14 (0.99, 1.30)	1.14 (0.99, 1.31)	192	1.08 (0.89, 1.30)	1.08 (0.89, 1.31)
0-<25%	0, 0.17	9873	61	1.0 (reference)	1.0 (reference)	30	1.0 (reference)	1.0 (reference)
25-<50%	0.17, 0.29	14869	103	1.17 (0.84, 1.62)	1.17 (0.85, 1.62)	53	1.17 (0.74, 1.86)	1.16 (0.73, 1.84)
50-<75%	0.29, 0.43	14959	107	1.25 (0.89, 1.76)	1.25 (0.89, 1.76)	55	1.21 (0.75, 1.96)	1.20 (0.74, 1.94)
>=75%	0.43, 2.94	14929	118	1.50 (1.03, 2.18)	1.50 (1.03, 2.19)	54	1.24 (0.72, 2.11)	1.23 (0.72, 2.11)
P trend[7]				0.03	0.03		0.52	0.53
**Processed meat nitrite**	Log2	54630	389	1.00 (0.97, 1.03)	1.00 (0.97, 1.03)	192	0.99 (0.95, 1.04)	0.99 (0.95, 1.04)
0-<25%	0, 0	12809	91	1.0 (reference)	1.0 (reference)	45	1.0 (reference)	1.0 (reference)
25-<50%	0.01, 0.06	14182	99	0.98 (0.73, 1.30)	0.98 (0.74, 1.30)	52	1.03 (0.69, 1.54)	1.02 (0.68, 1.52)
50-<75%	0.06, 0.14	13782	110	1.16 (0.88, 1.53)	1.16 (0.88, 1.54)	52	1.09 (0.73, 1.63)	1.07 (0.72, 1.61)
>=75%	0.14, 2.78	13857	89	0.95 (0.7, 1.28)	0.94 (0.69, 1.28)	43	0.90 (0.58, 1.38)	0.89 (0.57, 1.38)

P trend[7]	0.75	0.71	0.53	0.52

[1]CWS concentrations represent 15-year average (1990-2005) concentrations of arsenic (µg/L), uranium (µg/L), nitrate-N (mg/L), and TTHM (µg/L).
[2]Residential duration at the enrollment address was determined based on the residential history and self-reported information about duration at the current home at questionnaire 4. Mean follow-up=19.1 years, total person-years: 1,045,277. For uranium, mean follow-up=19.1 years, total person-years: 982,731.7.
[3]Dietary nitrite and nitrate (g/day) and total caloric intake (kilocalories/day) were computed based on the food frequency questionnaire at survey 1 and from published estimates. Zero values were imputed as the lowest non-zero estimate / 10; zero values were imputed as follows: total nitrate (0.005 mg/day), total nitrite (0.0005 mg/day), plant nitrite (0.0005 mg/day), animal nitrite (9.7E-6 mg/day), processed meat nitrite (0.0005 mg/day).
[4]Model 1 was adjusted for baseline age (years) + baseline age^2 + natural log transformed total daily caloric intake (kilocalories/day).
[5]Model 2 = model 1 + BMI category (<25 kg/m^2, 25-<30 kg/m^2, >30 kg/m^2, or missing), menopause status (pre-, peri/post-menopause, or missing), live births (yes/no/missing), oral contraceptive use (never/ever/missing).
[6]P trend for interaction was evaluated using the likelihood ratio test Chi squared test statistic.
[7]P trend was evaluated using the median of each quantile.

Table S13. Hazard ratios (95% CI) for all epithelial ovarian carcinomas and high grade serous ovarian cancers per doubling and categories of community water system (CWS) gross alpha exposures, among California Teachers Study participants. Long-term average CWS exposures were linked to the address at enrollment, and analyses were restricted to participants with a residential duration at enrollment ≥ 10 years (N=59,881)[1] for the main analyses; other analyses groups described below.

			All Epithelial Carcinomas			High-grade Serous Histotype		
**Gross alpha**	**Concentration**	**N**	**N cases**	**HR (95% CI) Model 1[2]**	**HR (95% CI) Model 2[3]**	**N Cases**	**HR (95% CI) Model 1[2]**	**HR (95% CI) Model 2[3]**
				**Main analyses participants, N=59,881**				
**15-year average (1990-2005)[1]**								
**Log2**		59881	413	1.11 (1.01, 1.22)	1.11 (1.01, 1.22)	199	1.07 (0.93, 1.23)	1.07 (0.93, 1.23)
**Per quantile**	**Range**							
0-<25%	0.06, 1.16	59881	413	1.0 (reference)	1.0 (reference)	199	1.0 (reference)	1.0 (reference)
25-<50%	1.17, 2.15	13821	85	1.10 (0.82, 1.47)	1.09 (0.82, 1.46)	41	1.16 (0.77, 1.76)	1.14 (0.76, 1.73)
50-<75%	2.15, 3.49	15271	98	1.27 (0.96, 1.68)	1.26 (0.96, 1.67)	50	1.34 (0.90, 2.00)	1.32 (0.89, 1.97)
75-<90%	3.49, 4.62	15767	118	1.33 (0.97, 1.83)	1.31 (0.96, 1.80)	60	1.18 (0.74, 1.89)	1.18 (0.74, 1.89)
>=90%	4.63, 60.57	8738	70	1.15 (0.79, 1.66)	1.14 (0.79, 1.65)	30	1.02 (0.59, 1.77)	1.01 (0.58, 1.75)
P trend[4]				0.15	0.17		0.74	0.75
**% years ≥ MCL/2[5]**								
0%		49083	329	1.0 (ref)	1.0 (ref)	159	1.0 (ref)	1.0 (ref)
>0-≤10%		5938	50	1.25 (0.93, 1.69)	1.24 (0.92, 1.68)	23	1.19 (0.77, 1.85)	1.19 (0.77, 1.84)
>10%		4860	34	1.08 (0.76, 1.54)	1.08 (0.76, 1.53)	17	1.12 (0.68, 1.84)	1.11 (0.67, 1.84)
**By urbanicity[6]**	**IQR**							
Non-metropolitan	2.06 (1.24,3.22)	18775	128	1.16 (0.97, 1.40)	1.17 (0.97, 1.40)	56	1.34 (1.01, 1.78)	1.34 (1.01, 1.78)
Metropolitan	2.18 (1.17,3.68)	40777	282	1.10 (0.98, 1.23)	1.09 (0.97, 1.22)	141	1.01 (0.86, 1.18)	1.00 (0.86, 1.18)
15-year average (1990-2005)[1]				**Participants censored on or after 2005, N=50,284[7]**				
**Log2**		50284	246	1.09 (0.96, 1.24)	1.09 (0.96, 1.23)	119	1.03 (0.86, 1.23)	1.02 (0.86, 1.23)
**Per quantile**	**Range**							
0-<25%	0.06, 1.16	11564	48	1.0 (reference)	1.0 (reference)	26	1.0 (reference)	1.0 (reference)
25-<50%	1.17, 2.15	12858	62	1.22 (0.83, 1.77)	1.22 (0.83, 1.77)	29	1.05 (0.62, 1.79)	1.04 (0.61, 1.76)
50-<75%	2.15, 3.49	13244	72	1.37 (0.95, 1.97)	1.36 (0.95, 1.97)	37	1.30 (0.79, 2.15)	1.28 (0.78, 2.12)
75-<90%	3.49, 4.62	7325	41	1.39 (0.91, 2.10)	1.36 (0.89, 2.06)	17	1.06 (0.58, 1.96)	1.05 (0.57, 1.93)
>=90%	4.63, 60.57	5293	23	1.10 (0.67, 1.81)	1.10 (0.67, 1.80)	10	0.89 (0.43, 1.84)	0.87 (0.42, 1.81)
P trend[4]				0.39	0.43		0.98	0.96

Cumulative average lagged 5 years		Time-varying exposures, N=85,684[8]					
**Log2**		566	1.05 (0.96, 1.14)	1.05 (0.96, 1.14)	283	1.02 (0.90, 1.14)	1.01 (0.90, 1.14)
**Per quantile**	**Range**						
0-<25%	0.04,1.18	139	1.0 (reference)	1.0 (reference)	71	1.0 (reference)	1.0 (reference)
25-<50%	1.18,2.25	134	1.02 (0.80, 1.29)	1.02 (0.80, 1.29)	70	1.06 (0.76, 1.47)	1.05 (0.75, 1.46)
50-<75%	2.25,3.53	147	1.06 (0.84, 1.34)	1.06 (0.84, 1.34)	74	1.05 (0.76, 1.46)	1.05 (0.76, 1.45)
75-<90%	3.53,4.63	100	1.26 (0.97, 1.63)	1.25 (0.97, 1.63)	52	1.28 (0.89, 1.84)	1.28 (0.89, 1.83)
>=90%	4.63,113.58	46	0.82 (0.59, 1.15)	0.82 (0.58, 1.14)	16	0.56 (0.33, 0.97)	0.56 (0.32, 0.96)
P trend[4]			0.99	0.98		0.34	0.34

[1]CWS concentrations represent 15-year average (1990-2005) concentrations of gross alpha (pCi/L). Residential duration at the enrollment address was determined based on the residential history and self-reported information about duration at the current home at questionnaire 4. Mean follow-up=19.0 years, total person-years: 1,139,582.
[2]Model 1 was adjusted for baseline age (years) + baseline age^2.
[3]Model 2 = model 1 + BMI category (<25 kg/m^2, 25-<30 kg/m^2, >30 kg/m^2, or missing), menopause status (pre-, peri/post-menopause, or missing), live births (yes/no/missing), oral contraceptive use (never/ever/missing).
[4]P trend was evaluated using the median of each quantile.
[5]The percentage of years of CWS gross alpha exceeding ½ of the maximum contaminant level (MCL) was calculated as the number of years that average annual concentrations exceeded ½ the MCL / the number of years of monitoring data reported for each contaminant and modeled as continuous variables, and comparing 0% (reference), >0-≤10%, and >10% of years.
[6]For non-metropolitan participants, mean follow-up=18.9 years, total person-years: 355,743. For metropolitan participants, mean follow-up=19.1 years, total person-years: 777113.8. Analyses were modeled using log2 transformed concentrations.
[7]Analyses were restricted to participants censored on or after Jan 1, 2005 (participants who were censored prior to 2005 were excluded). Mean follow-up=12.7 years, total person-years=641,119.9. Person time was re-calculated so that follow-up time started on Jan 1, 2005, and age was re-calculated to reflect age on Jan 1, 2005 (baseline age + years between the participant start date and Jan 1, 2005). Model 1 was adjusted for age in 2005 (years) + (age in 2005)^2. Model 2 = model 1 + BMI category (<25 kg/m^2, 25-<30 kg/m^2, >30 kg/m^2, or missing), menopause status (pre-, peri/post-menopause, or missing), live births (yes/no/missing), oral contraceptive use (never/ever/missing).
[8]For time-varying exposures, CWS were linked by addresses from the residential history and represent cumulative average exposures lagged 5 years. Total person-years=1,563,033.

Table S14. Hazard ratios (95% CI) for all epithelial ovarian carcinomas and high grade serous ovarian cancers per doubling of community water system (CWS) exposures, and per interquartile range increase in the contaminant mixture, among California Teachers Study participants, excluding participants censored prior to 2005. CWS exposures[1] were linked to the address at enrollment. Analyses were restricted to participants with a residential duration at enrollment ≥ 10 years[2] with follow-up beginning in 2005.[3]

			All Epithelial Carcinomas			High-grade Serous Histotype		
	CWS Concentration	N	N cases	HR (95% CI) Model 1[4]	HR (95% CI) Model 2[5]	N Cases	HR (95% CI) Model 1[4]	HR (95% CI) Model 2[5]
**Nitrate-N**	Log2	50284	246	1.03 (0.97, 1.1)	1.03 (0.97, 1.1)	119	1.07 (0.98, 1.17)	1.06 (0.97, 1.17)
0-<25%	0.01, 0.22	12351	53	1.0 (reference)	1.0 (reference)	23	1.0 (reference)	1.0 (reference)
25-<50%	0.22, 0.56	12522	60	1.12 (0.77, 1.61)	1.11 (0.77, 1.61)	27	1.16 (0.66, 2.02)	1.15 (0.66, 2)
50-<75%	0.57, 2.24	12833	72	1.35 (0.94, 1.92)	1.35 (0.94, 1.92)	33	1.42 (0.84, 2.42)	1.4 (0.82, 2.39)
75-<90%	2.25, 3.74	7538	40	1.27 (0.84, 1.92)	1.26 (0.84, 1.9)	24	1.76 (0.99, 3.12)	1.73 (0.97, 3.07)
>=90%	3.77, 9.62	5040	21	1.02 (0.61, 1.69)	1.02 (0.62, 1.7)	12	1.34 (0.67, 2.7)	1.31 (0.65, 2.64)
P trend[6]				0.86	0.87		0.21	0.24
**Uranium**	Log2	47319	234	1.06 (0.97, 1.15)	1.05 (0.97, 1.15)	114	1.04 (0.92, 1.18)	1.04 (0.92, 1.17)
0-<25%	0.09, 1.35	11573	47	1.0 (reference)	1.0 (reference)	25	1.0 (reference)	1.0 (reference)
25-<50%	1.35, 3.79	12132	63	1.33 (0.91, 1.94)	1.33 (0.91, 1.94)	32	1.27 (0.75, 2.14)	1.26 (0.75, 2.13)
50-<75%	3.8, 6.46	11840	66	1.44 (0.99, 2.09)	1.43 (0.98, 2.07)	29	1.19 (0.7, 2.03)	1.17 (0.69, 2)
75-<90%	6.54, 10.96	6817	32	1.18 (0.75, 1.85)	1.18 (0.75, 1.85)	15	1.04 (0.55, 1.97)	1.03 (0.54, 1.96)
>=90%	11.15, 100.93	4957	26	1.37 (0.85, 2.21)	1.35 (0.84, 2.18)	13	1.29 (0.66, 2.52)	1.26 (0.65, 2.47)
P trend[6]				0.36	0.39		0.68	0.72
**Arsenic**		50284	246	1.05 (0.94, 1.17)	1.05 (0.94, 1.16)	119	1.07 (0.92, 1.24)	1.06 (0.91, 1.24)
0-<25%	0.01, 0.59	11749	56	1.0 (reference)	1.0 (reference)	28	1.0 (reference)	1.0 (reference)
25-<50%	0.59, 0.99	12971	63	1.04 (0.73, 1.5)	1.03 (0.72, 1.48)	27	0.9 (0.53, 1.52)	0.89 (0.53, 1.52)
50-<75%	0.99, 1.79	12436	61	1.05 (0.73, 1.51)	1.04 (0.72, 1.5)	28	0.96 (0.57, 1.63)	0.95 (0.56, 1.61)
75-<90%	1.8, 3.09	7487	35	1.03 (0.67, 1.57)	1.02 (0.67, 1.56)	21	1.24 (0.7, 2.18)	1.21 (0.69, 2.14)
>=90%	3.1, 36.55	5641	31	1.19 (0.77, 1.84)	1.16 (0.75, 1.8)	15	1.15 (0.61, 2.16)	1.14 (0.61, 2.13)
P trend[6]				0.48	0.53		0.41	0.44
**TTHM**		50284	246	1.01 (0.97, 1.05)	1.01 (0.97, 1.05)	119	1.02 (0.96, 1.08)	1.02 (0.96, 1.08)
0-<25%	0, 0.31	12508	58	1.0 (reference)	1.0 (reference)	27	1.0 (reference)	1.0 (reference)
25-<50%	0.31, 4.55	12435	60	1.02 (0.71, 1.46)	1.02 (0.71, 1.46)	28	1.02 (0.6, 1.74)	1.03 (0.61, 1.75)
50-<75%	4.56, 20.82	12764	60	0.99 (0.69, 1.42)	0.99 (0.69, 1.42)	34	1.2 (0.73, 1.99)	1.21 (0.73, 2.01)
75-<90%	20.84, 30.86	7433	38	1.06 (0.7, 1.59)	1.06 (0.7, 1.6)	15	0.9 (0.48, 1.68)	0.91 (0.48, 1.71)
>=90%	30.94, 108.66	5144	30	1.25 (0.8, 1.94)	1.26 (0.81, 1.95)	15	1.34 (0.71, 2.52)	1.35 (0.72, 2.55)
P trend[6]				0.35	0.33		0.57	0.54

**Contaminant mixture**[7]	47319	241	1.33 (0.86, 2.06)	1.33 (0.86, 2.06)	115	1.82 (0.97, 3.41)	1.81 (0.96, 3.39)

[1]CWS concentrations represent 15-year average (1990-2005) concentrations of arsenic (µg/L), uranium (µg/L), nitrate-N (mg/L), and TTHM (µg/L).
[2]Residential duration at the enrollment address was determined based on the residential history and self-reported information about duration at the current home at questionnaire 4.
[3]Mean follow-up=12.8 years, total person-years=641,119.9. For uranium, total person-years=603,545.2. Person time was re-calculated so that follow-up time started on Jan 1, 2005, and age was re-calculated to reflect age on Jan 1, 2005 (baseline age + years between the participant start date and Jan 1, 2005).
[4]Model 1 was adjusted for age in 2005 (years) + (age in 2005)^2.
[5]Model 2 = model 1 + BMI category (<25 kg/m^2, 25-<30 kg/m^2, >30 kg/m^2, or missing), menopause status (pre-, peri/post-menopause, or missing), live births (yes/no/missing), oral contraceptive use (never/ever/missing).
[6]P trend was evaluated using the median of each quantile.
[7]Concentrations were log2 transformed and subsequently divided by the IQR. The effect of the drinking water contaminant mixture was evaluated using quantile q-computation, and hazards ratios are interpreted per IQR increase.

Table S15. Hazard ratios (95% CI) for all epithelial ovarian carcinomas and high grade serous ovarian cancers per doubling of community water system (CWS) exposures, and per interquartile range increase in the contaminant mixture, among California Teachers Study participants with exposures < maximum contaminant levels (MCL). CWS exposures[1] were linked to the address at enrollment. Analyses were restricted to participants with a residential duration at enrollment ≥ 10 years[2], with long-term average nitrate, arsenic, uranium, gross alpha, and TTHM concentrations < corresponding MCLs.[3]

		All Epithelial Carcinomas			High-grade Serous Histotype		
	**N**	**N cases**	**HR (95% CI) Model 1[4]**	**HR (95% CI) Model 2[5]**	**N Cases**	**HR (95% CI) Model 1[4]**	**HR (95% CI) Model 2[5]**
Nitrate-N	59881	413	1.03 (0.98, 1.08)	1.03 (0.98, 1.08)	199	1.09 (1.02, 1.17)	1.09 (1.01, 1.17)
Uranium	56262	391	1.07 (1.00, 1.15)	1.07 (1.00, 1.14)	194	1.05 (0.95, 1.15)	1.05 (0.95, 1.15)
Arsenic	59362	408	1.04 (0.96, 1.14)	1.04 (0.95, 1.13)	197	1.08 (0.95, 1.22)	1.07 (0.95, 1.22)
TTHM	59845	413	1.01 (0.98, 1.04)	1.01 (0.98, 1.04)	199	1.01 (0.97, 1.06)	1.01 (0.97, 1.06)
**Contaminant mixture[6]**	55813	387	1.35 (0.96, 1.90)	1.34 (0.96, 1.89)	192	1.76 (1.09, 2.86)	1.75 (1.08, 2.84)

[1]CWS concentrations represent 15-year average (1990-2005) concentrations of arsenic (µg/L), uranium (µg/L), nitrate-N (mg/L), and TTHM (µg/L). No average concentrations of nitrate-N were above the MCL, so the nitrate-N (single contaminant model) results are identical to Table 2.
[2]Residential duration at the enrollment address was determined based on the residential history and self-reported information about duration at the current home at questionnaire 4.
[3]In the mixtures models (all contaminant averages < MCLs): N=55,813, mean follow-up =19.05 years, total person-years=1,062,970.
[4]Model 1 was adjusted for baseline age (years) + baseline age^2.
[5]Model 2 = model 1 + BMI category (<25 kg/m^2, 25-<30 kg/m^2, >30 kg/m^2, or missing), menopause status (pre-, peri/post-menopause, or missing), live births (yes/no/missing), oral contraceptive use (never/ever/missing).
[6]Concentrations were log2 transformed and subsequently divided by the IQR. The effect of the drinking water contaminant mixture was evaluated using quantile q-computation, and hazards ratios are interpreted per IQR increase.

Figure S1. Inclusion and exclusion criteria of community water system (CWS) exposures and ovarian cancer risk in the California Teachers Study. CA=California. TTHM=total trihalomethanes.

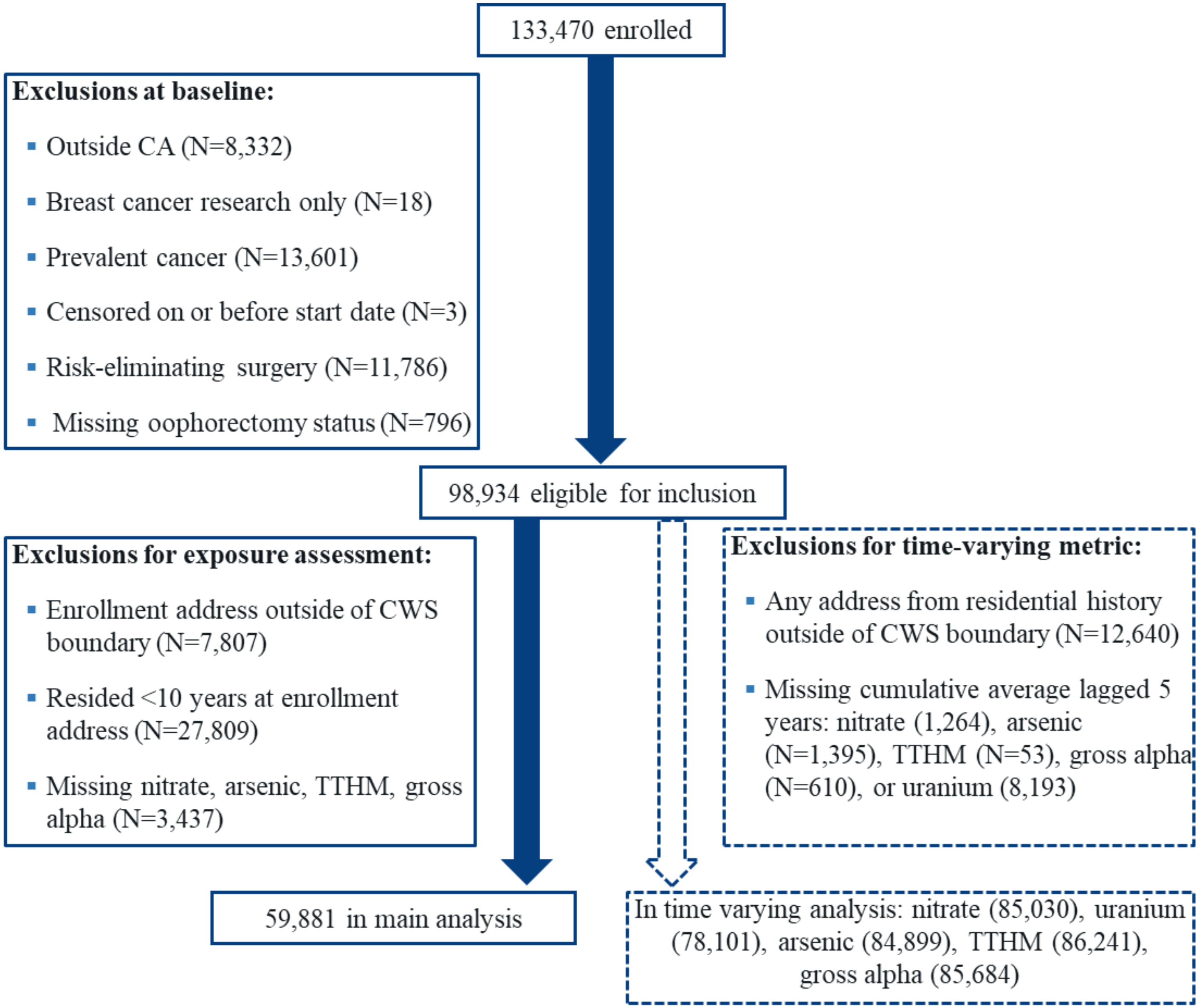

Figure S2. Map of community water systems (CWS) serving California Teachers Study participants in California. CWS that serve > 5 participants are shown.

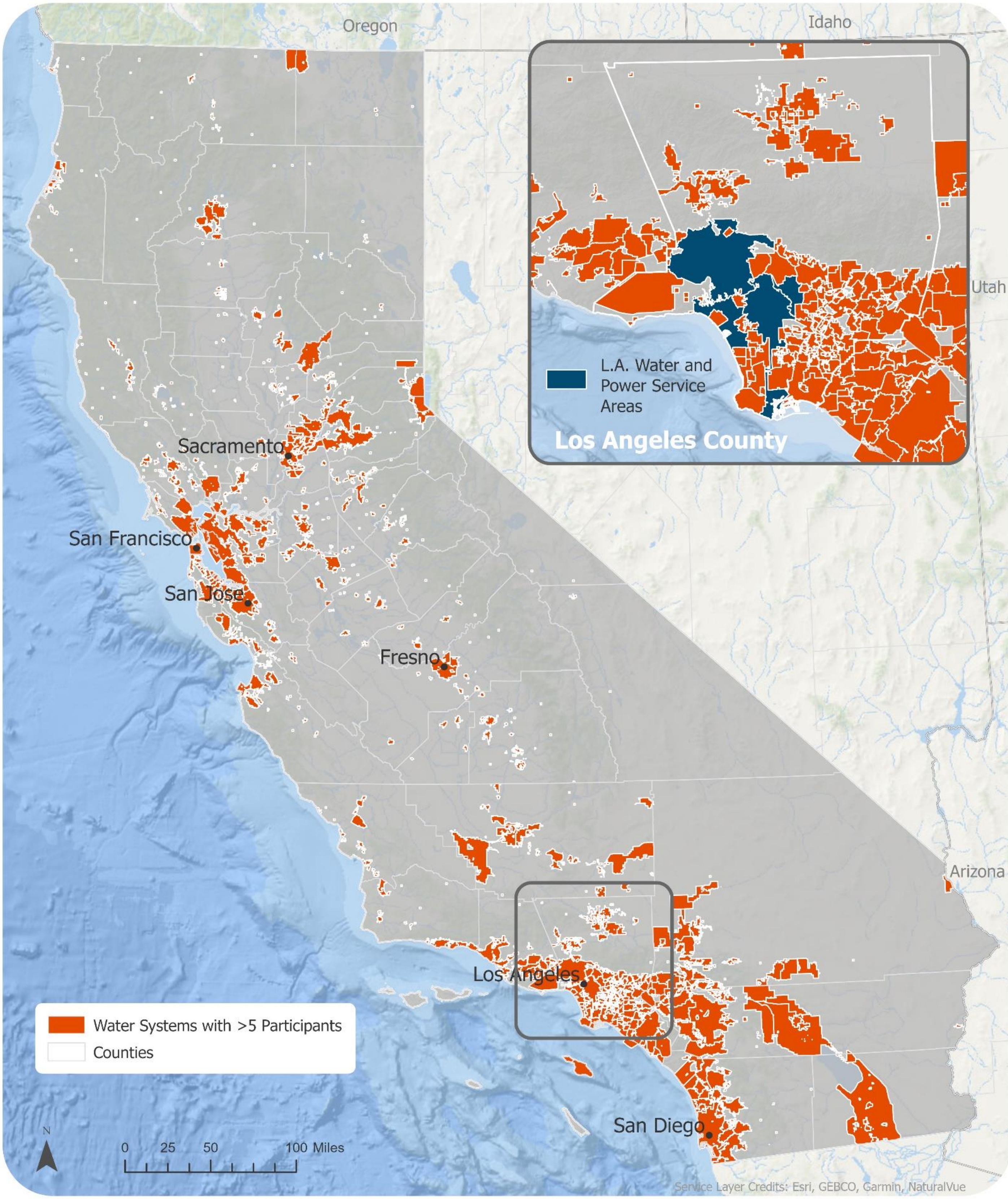

Figure S3. Spearman correlation coefficients of 15-year average (1990-2005) community water system (CWS) arsenic, uranium, gross alpha, nitrate-nitrogen (N), and total trihalomethanes (TTHM) concentrations among California Teachers Study (CTS) participants. Positive correlations are shown in blue and negative correlations are shown in red.

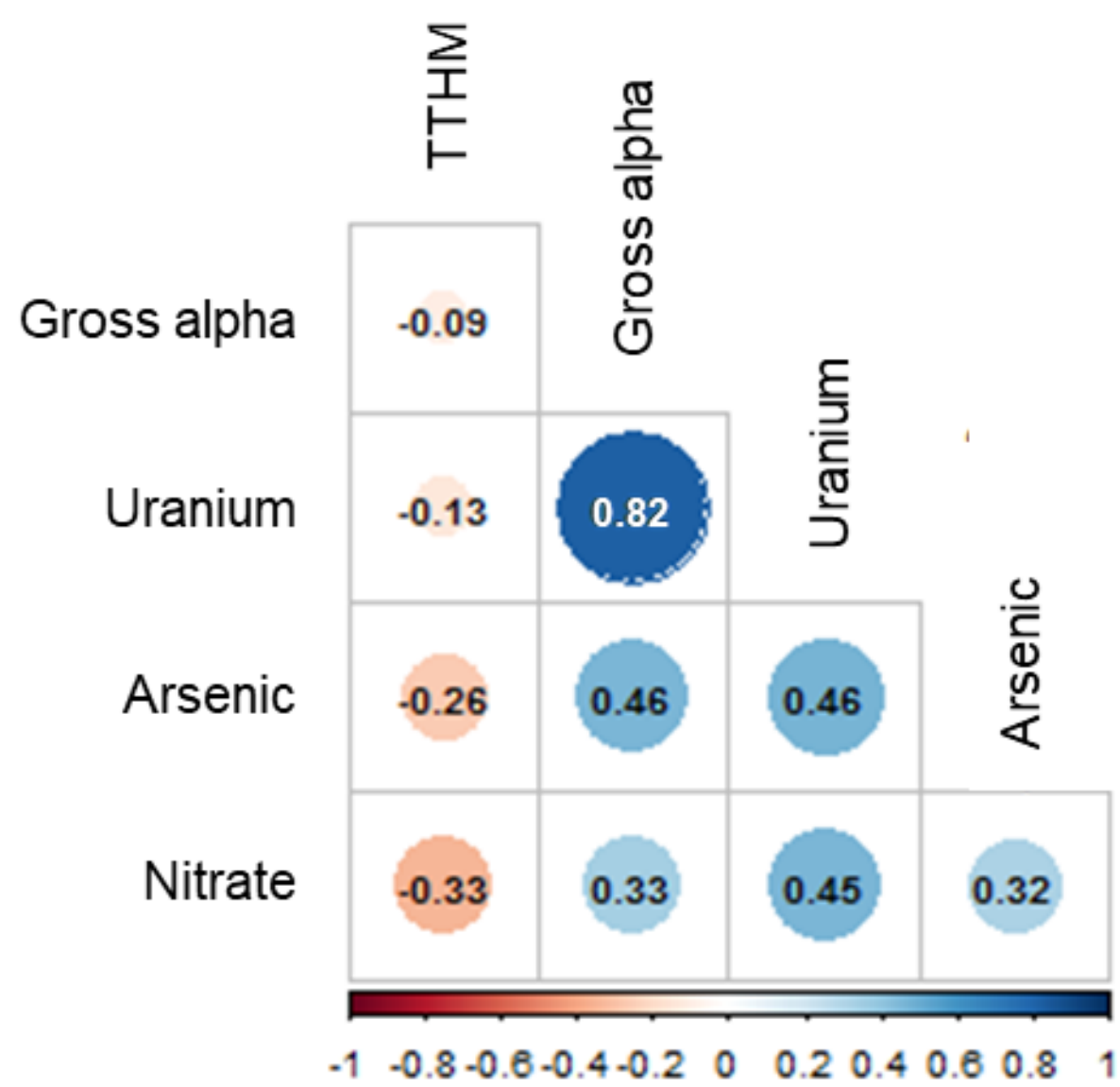

Figure S4. Spearman correlation coefficients of 15-year average (1990-2005) community water system (CWS) arsenic, uranium, gross alpha, nitrate-nitrogen (N), and total trihalomethanes (TTHM) concentrations among California Teachers Study (CTS) participants, among non-metropolitan *(left)* and metropolitan participants *(right)*. Positive correlations are shown in blue and negative correlations are shown in red.

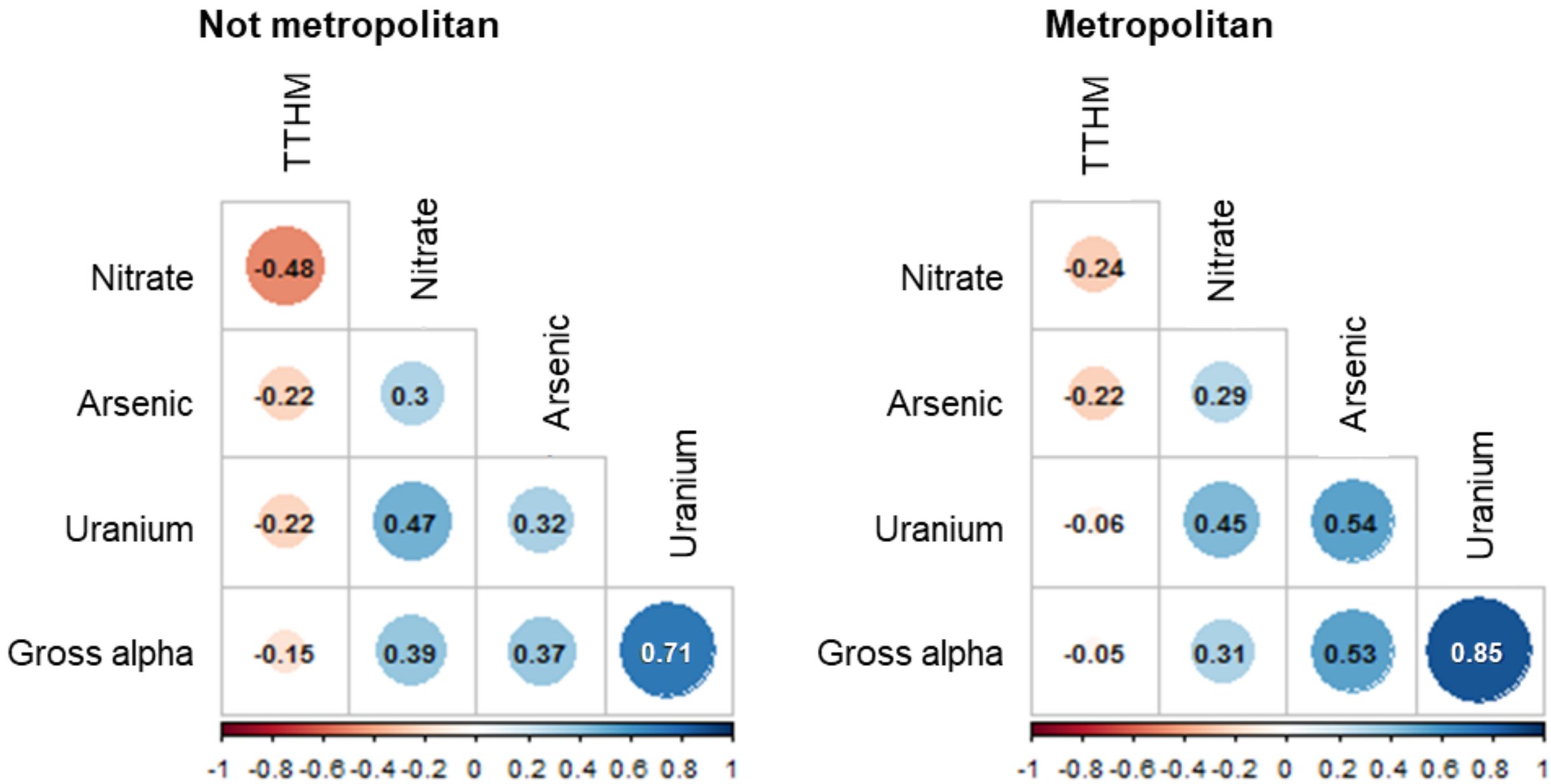

References

1. United States Environmental Protection Agency. Arsenic and clarifications to compliance and new source monitoring rule: A quick reference guide. Accessed Dec 15, 2022. https://denr.sd.gov/des/dw/PDF/Quick%20Reference%20Guides/quickguide.pdf
2. United States Environmental Protection Agency. Arsenic Rule Compliance for Community Water System Owners and Operators. Accessed Dec 15, 2022. https://www.epa.gov/dwreginfo/arsenic-rule-compliance-community-water-system-owners-and-operators
3. United States Environmental Protection Agency. Radionuclides Rule. Accessed Dec 15, 2022. https://www.epa.gov/dwreginfo/radionuclides-rule#:~:text=The%20new%20MCL%20for%20uranium%20is%2030%20%C2%B5g%2FL.
4. United States Environmental Protection Agency. Stage 1 and Stage 2 Disinfectants and Disinfection Byproducts Rules. Accessed 7 July, 2023. https://www.epa.gov/dwreginfo/stage-1-and-stage-2-disinfectants-and-disinfection-byproducts-rules
5. United States Environmental Protection Agency. Chemical Contaminant Rules. Accessed Dec 15, 2022. https://www.epa.gov/dwreginfo/chemical-contaminant-rules
6. The Standardized Monitoring Framework: A Quick Reference Guide. (2004).
7. Hurley SE, Reynolds, P., Goldberg, D. E., Hertz, A., Anton-Culver, H., Bernstein, L., ... & Horn-Ross, P. L. Residential mobility in the California Teachers Study: implications for geographic differences in disease rates. *Social science & medicine*. 2005;60(7):1547-1555.
8. Spaur M, Medgyesi, D. N., Bangia, K., Madrigal, J. M., Hurwitz, L. M., Beane Freeman, L. E., Fisher, J. A., Spielfogel, E. S., Lacey Jr., J. V., Sanchez, T., Jones, R. R., Ward, M. H. Drinking water source and exposure to regulated water contaminants in the California Teachers Study cohort. *Journal of Exposure Science & Environmental Epidemiology*. 2024:1-12.
